# METTL3 facilitates renal cell carcinoma progression by PLOD2 m^6^A-methylation under prolonged hypoxia

**DOI:** 10.1038/s41419-023-06411-w

**Published:** 2024-01-17

**Authors:** Yimeng Chen, Yichen He, Zhengsheng Li, Nan Zhang, Cuixing Zhou, Xiaozhou He, Dong Xue

**Affiliations:** Department of Urology, The Third Affiliated Hospital of Soochow University, Changzhou, 213003 Jiangsu China

**Keywords:** Renal cell carcinoma, Methylation

## Abstract

N6-methyladenosine (m^6^A) is the most prevalent reversible modification in eukaryotic mRNA, and it plays a critical role in tumor progression. The purpose of this study was to investigate the function and regulatory mechanisms of the methyltransferase METTL3 in renal cell carcinoma (RCC). METTL3 expression was upregulated and predicted a poor prognosis in patients with advanced RCC. METTL3 facilitated the proliferation, migration, and invasion of RCC cells, depending on its methylase activity. METTL3 positively regulated the expression of PLOD2, and both genes were triggered under prolonged hypoxia. Mechanistically, hypoxia-induced the binding of HIF-1α to the METTL3 promoter, which enhanced its transcriptional activity. METTL3-mediated m^6^A modifications of PLOD2 mRNA at 3’UTR region, promoting the translation of PLOD2 protein. Furthermore, silencing METTL3 impaired RCC progression in vitro. In vivo, administration of highly potent and selective METTL3 inhibitor STM2457 showed anti-tumor effects, whereas AAV9-mediated re-transduction of PLOD2 largely abolished the above phenomenon in a subcutaneous mouse model. These findings reveal that hypoxia and HIF-driven METTL3 transcription promote RCC progression by increasing PLOD2 expression in an m^6^A-dependent manner, suggesting that METTL3 may serve as a novel pharmaceutical intervention for RCC.

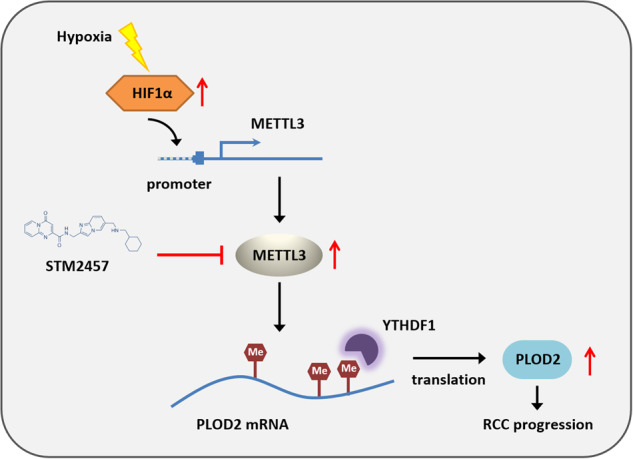

## Introduction

Renal cell carcinoma (RCC) is one of the most common malignant cancers worldwide, with a relatively high mortality among cancers of the urinary system [1]. According to Global Cancer Statistics, there were >431,000 patients with RCC and 179,000 deaths globally in 2020, and the incidence of RCC is still increasing in recent years [2]. Thus, it is of great importance to investigate the underlying mechanism and to develop potential therapeutic targets for advanced RCC.

Emerging evidence suggests that abnormal expression of oncogenes or tumor-suppressor genes plays critical roles in cancer progression. N6-methyladenosine (m^6^A) modification is the most prevalent internal mRNA modification in eukaryote cells. It regulates gene expression by affecting the stability, localization, transportation, or translation of target mRNAs [3]. The process of m^6^A modification is reversible, facilitated by a methyltransferase complex (known as m^6^A ‘writers’), removed by m^6^A demethylases (known as m^6^A ‘erasers’), and executed by another set of m^6^A-binding proteins as ‘readers’ that mediate specific functions of methylated transcripts [4, 5]. Methyltransferase 3 (METTL3) is the key component of the m^6^A methyltransferase complex that catalyzes the methylation of target transcripts. Aberrant m^6^A modification profiles and abnormal expression of METTL3 have been associated with the initiation and progression of different types of cancers [5–8]. In our previous study, we profiled the first m^6^A transcriptome-wide map of human RCC and discovered highly diverse m^6^A modification patterns between RCC tissues and healthy controls [9]. However, the specific role of METTL3-mediated m^6^A modification in RCC progression needs to be further investigated.

Oxygen deprivation and intratumoral hypoxia exert a critical impact on the progression of solid tumors, including advanced RCC. The median partial pressure of oxygen (pO_2_) is 10 *mm Hg* in RCC tissue compared with 31 *mm Hg* in normal kidney tissue, indicating distinctly poorer oxygenation within tumors [10]. Hypoxic condition can stimulate various pathways in cancer cells, leading to dysregulation of genes and facilitating the progression of RCC. Procollagen-lysine, 2-oxoglutarate 5-dioxygenase 2 (PLOD2), a member of PLOD family (PLOD1, PLOD2, and PLOD3), mediates the formation of stabilized collagen cross-links [11]. PLOD2 has oncogenic roles and acts as a prognostic biomarker in several types of cancers [12, 13]. It leads to increased collagen deposition, extracellular matrix (ECM) remodeling, and metastatic potential of cancer cells. Emerging evidence suggests that PLOD2 expression can be induced under hypoxic conditions and thus promote tumor progression [14, 15], but the molecular mechanism has not been elucidated. Indeed, our recent study and those of others reported a dramatic increase in PLOD2 expression, along with elevated m^6^A enrichment on its transcript in RCC samples [9, 16], indicating that PLOD2 expression can be post-transcriptionally regulated by m^6^A modifications.

In this study, we demonstrated the importance of HIF/METTL3/PLOD2 axis in RCC progression. METTL3 is upregulated in advanced RCC patients and enhances PLOD2 expression in a m^6^A-dependent manner. Hypoxic microenvironment promotes METTL3 and PLOD2 expression by inducing transcriptional factor HIF. Therefore, targeting METTL3 and related pathways may be a novel pharmaceutical intervention for RCC patients.

## Results

### Upregulated METTL3 predicts poor survival in RCC patients

**Fig. 1:**
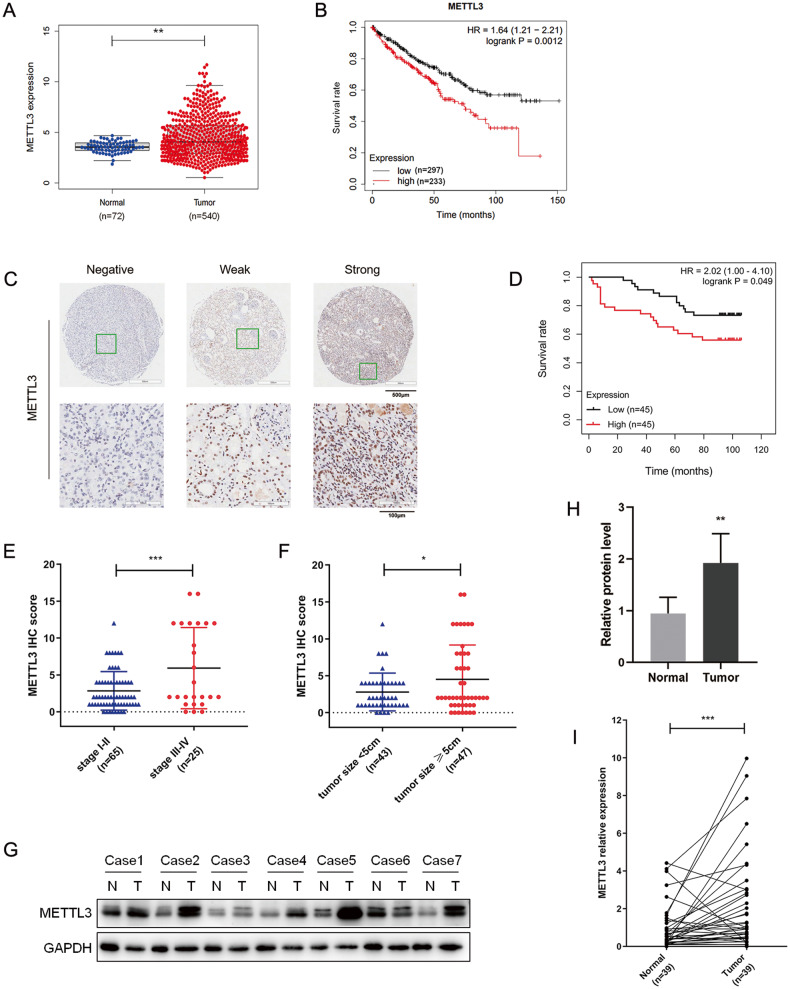
Upregulated METTL3 predicts poor survival in RCC patients. **A** METTL3 expression in the normal tissues (*n* = 72) and RCC samples (*n* = 540) in the TCGA cohort. **P = 0.0020. **B** Kaplan–Meier survival curve with log-rank test was applied for prognostic evaluation in a total of 530 RCC patients from the Kaplan–Meier Plotter dataset. **C** Representative images of IHC staining on a tissue microarray with negative, weak or strong METTL3 expression. **D** Kaplan–Meier survival curve was plotted in human RCC samples with high (*n* = 45) or low (*n* = 45) METTL3 IHC scores. **E** METTL3 IHC scores in RCC tissues with different disease stages (stage I–II vs. stage III–IV). ***P = 0.0005. **F** METTL3 IHC scores in RCC tissues with different tumor size (<5 cm vs. ≥5 cm). *P = 0.0347. Data were represented as mean ± S.D. **G**–**I** Increased METTL3 expression in RCC tissues compared to their adjacent normal tissues was verified by Western blotting and RT-qPCR assay in our RCC cohort. N, normal; T, tumor. **P = 0.0011, ***P = 0.0009.

To explore the expression profile of METTL3 in RCC, the Cancer Genome Atlas (TCGA) data was utilized. Upregulation of METTL3 was observed in TCGA RCC samples (*n* = 540) compared with normal controls (*n* = 72) (*P* = 0.0020, Fig. 1A). Survival analysis identified an association between high METTL3 expression and short overall survival time in 530 RCC patients from the Kaplan–Meier Plotter dataset (www.kmplot.com) (*P* = 0.0012, Fig. 1B). Immunohistochemistry (IHC) staining was performed on tissue microarray (TMA) containing 90 paired RCC specimens and adjacent normal renal epithelial tissues with long-term clinical follow-up (Fig. 1C). METTL3 expression was recorded by assessment of positive cells and staining intensity. Individuals were assigned to two subgroups according to the median METTL3 IHC staining score. Kaplan–Meier survival analysis showed that patients with high METTL3 expression had shorter overall survival time than those with low METTL3 expression (*P* = 0.0049, Fig. 1D), which was consistent with the result from TCGA dataset. The subsequent analysis further revealed higher METTL3 IHC scores in tumors with advanced disease stages (*P* = 0.0005, Fig. 1E) and larger size (*P* = 0.0347, Fig. 1F). The results from our RCC cohort consists of 39 pairs of RCC tissues and their adjacent normal tissues also uncovered a significantly increased expression of METTL3 in RCC tissues, as determined by western blotting and RT-qPCR assay (Fig. 1G–I). Together, these results suggest that METTL3 is elevated in RCC and predicts poor survival of RCC patients.

### METTL3 promotes RCC progression dependent on its methylase activity

**Fig. 2:**
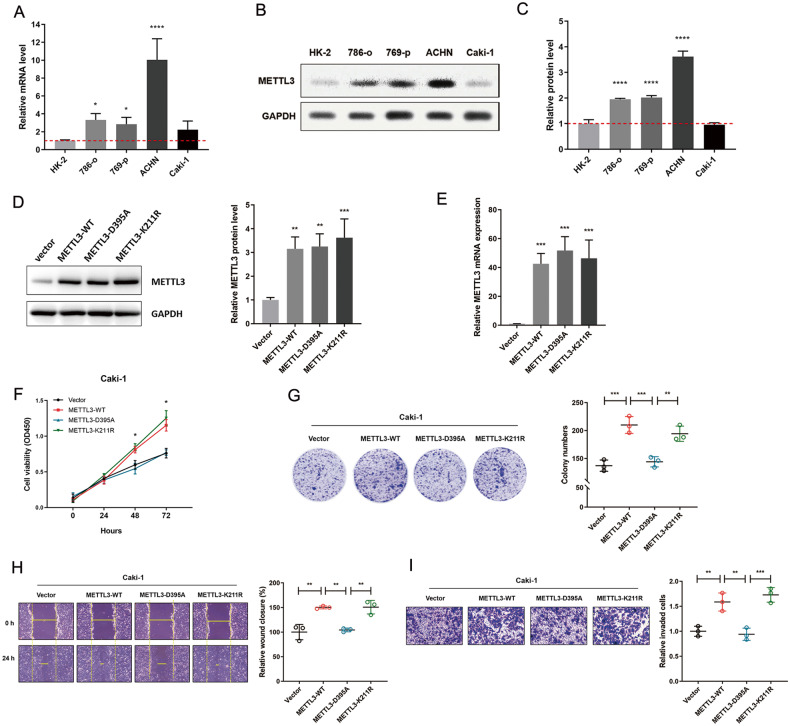
METTL3 promotes RCC progression dependent on its methylase activity. **A** Relative mRNA levels of METTL3 in four RCC cell lines and normal renal epithelial cells (HK-2) were determined by RT-qPCR. *P = 0.0220 (left), 0.0470 (right), ****P < 0.0001. **B**, **C** Western blotting analysis of METTL3 protein levels in four RCC cell lines and normal renal epithelial cells (HK-2). ****P < 0.0001. **D** METTL3 protein levels in Caki-1 cells transfected with METTL3 over-expressing vectors. **P = 0.0032 (left), 0.0024 (right), ***P = 0.0009. **E** METTL3 mRNA expression in Caki-1 cells transfected with METTL3 over-expressing vectors. ***P = 0.0007, 0.0001, and 0.0004 (left to right). **F** Cell growth curve of Caki-1 cells transfected with METTL3 over-expressing vectors. METTL3-D395A vs. WT, METTL3-D395A vs. K211R, *P < 0.05. **G** Colony formation assay of Caki-1 cells transfected with METTL3 over-expressing vectors. ***P = 0.0004 (left), 0.0008 (right), **P = 0.0045. **H** Wound-healing assay of Caki-1 cells transfected with METTL3 over-expressing vectors. **P = 0.0019, 0.0033, and 0.0031 (left to right). **I** Cell invasion ability of Caki-1 cells transfected with METTL3 over-expressing vectors. **P = 0.0040 (left), 0.0022 (right), ***P = 0.0006. Data represent means ± S.D. of three independent experiments.

METTL3 expression was assessed in normal renal epithelial cells (HK-2) and several RCC cell lines (786-o, 769-p, ACHN, and Caki-1). Western blotting and RT-qPCR assays confirmed the higher expression of METTL3 in RCC cell lines than the normal renal epithelial cells (Fig. 2A–C). Then, Caki-1 cells with the lowest endogenous METTL3 expression level were transfected with different types of METTL3-overexpression vectors. In METTL3-D395A vector, the METTL3 catalytic residue was disrupted by a point mutation to abrogate its methylation activity [17]. METTL3-K211R vector, however, is an enzymatic activity-unrelated mutant that contains a single mutation of lysine at position 211 [18, 19]. The efficiency of overexpression was verified by western blotting and RT-qPCR (Fig. 2D, E). Overexpression of METTL3-D395A failed to promote cell proliferation compared to METTL3-WT and METTL3-K211R, as determined in CCK-8 assay (Fig. 2F) and colony formation assay (Fig. 2G). In addition, forced expression of METTL3-WT or METTL3-K211R promoted wound closure and invasive activity in Caki-1 cells, whereas reduction of wound closure and invasiveness was observed in METTL3-D395A cells (Fig. 2H, I). These results suggest that METTL3 enhances tumor cell proliferation and RCC metastatic capacity dependent on the expression levels and its methylase activity.

### Hypoxia induces HIF- and METTL3-dependent PLOD2 expression

**Fig. 3:**
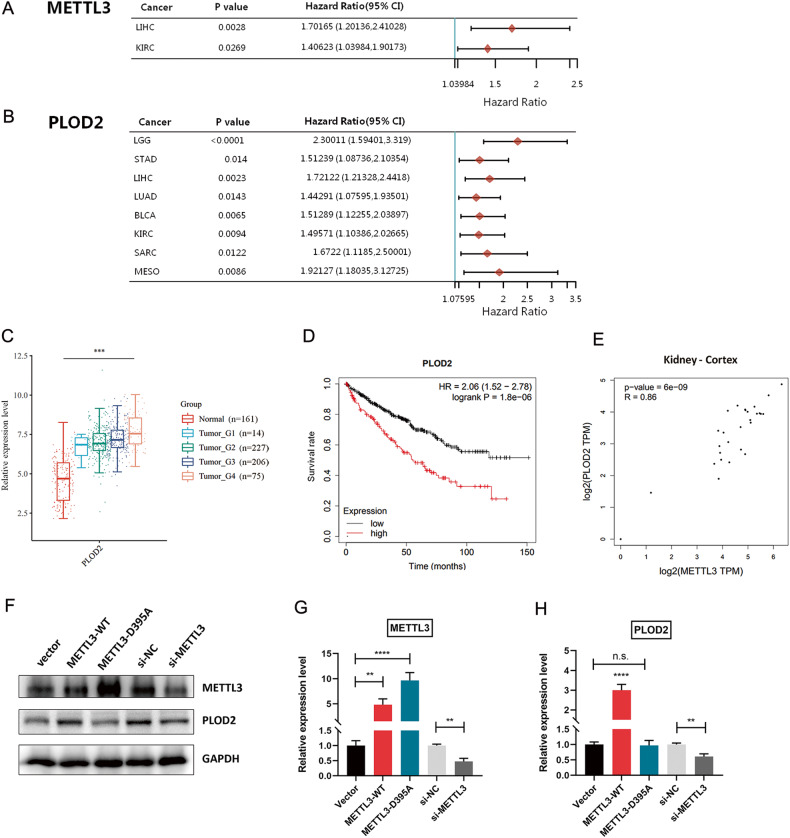
Pan-cancer analysis of METTL3 and PLOD2 gene. **A**, **B** Forest plot displayed the effects of METTL3 (**A**) and PLOD2 (**B**) on overall survival in multiple kinds of cancers. LIHC, Liver hepatocellular carcinoma; KIRC, Kidney renal clear cell carcinoma; LGG, Brain lower grade glioma; STAD, Stomach adenocarcinoma; LUAD, Lung adenocarcinoma; BLCA, Bladder urothelial carcinoma; SARC, Sarcoma; MESO, Mesothelioma. **C** PLOD2 expression level in a TCGA RCC cohort with different tumor grade. ***P < 0.001. **D** Kaplan–Meier survival curve with log-rank test was applied for prognostic evaluation in a total of 530 RCC patients from the Kaplan–Meier Plotter dataset. **E** Pearson correlation analysis between METTL3 and PLOD2 expression in the cortex of kidney. **F**–**H** Protein levels of METTL3 and PLOD2 in Caki-1 cells with METTL3-overexpression or depletion were determined by western blotting. **P = 0.0018, 0.0014, and 0.0025 (left to right), ****P < 0.0001. Data were represented as mean ± S.D. of three independent experiments.

Pan-cancer analysis tool was used to explore the relationships among gene expression and genesis of different kinds of cancers. Univariate cox-regression analysis showed that METTL3 is a risk factor of liver hepatocellular carcinoma (LIHC) and kidney renal clear cell carcinoma (KIRC) (Fig. 3A). Meanwhile, procollagen-lysine,2-oxoglutarate 5-dioxygenase 2 (PLOD2) gene has tumor-promoter effects in many types of cancers, including RCC (Fig. 3B). This is consistent with our previous report that RCC patients displayed much higher PLOD2 expression levels compared with healthy controls [9]. Further analysis of a large RCC cohort from the TCGA dataset revealed a significant elevation in PLOD2 expression with increasing tumor grade (Fig. 3C). Survival analysis identified a prominent association between high PLOD2 expression and short overall survival time in 530 RCC patients from the Kaplan–Meier Plotter dataset (*P* = 1.8e-06, Fig. 3D). Moreover, METTL3 could positively regulate PLOD2 expression in RCC, which was confirmed by gene expression profiling interactive analysis (GEPIA2) (*P* = 6e-09, Fig. 3E). Ectopic METTL3 expression and METTL3 depletion were used to confirm the role of METTL3 on PLOD2 expression induction in different cell lines including Caki-1 and ACHN cells (Fig. 3F–H and Supplementary Fig. 1A–C). In addition, METTL3-D395A mutant failed to induce PLOD2 protein levels, even with a high transfection dose (3 μg/well) or an extended duration (72 h after transfection) (Supplementary Fig. 1D–I).

**Fig. 4:**
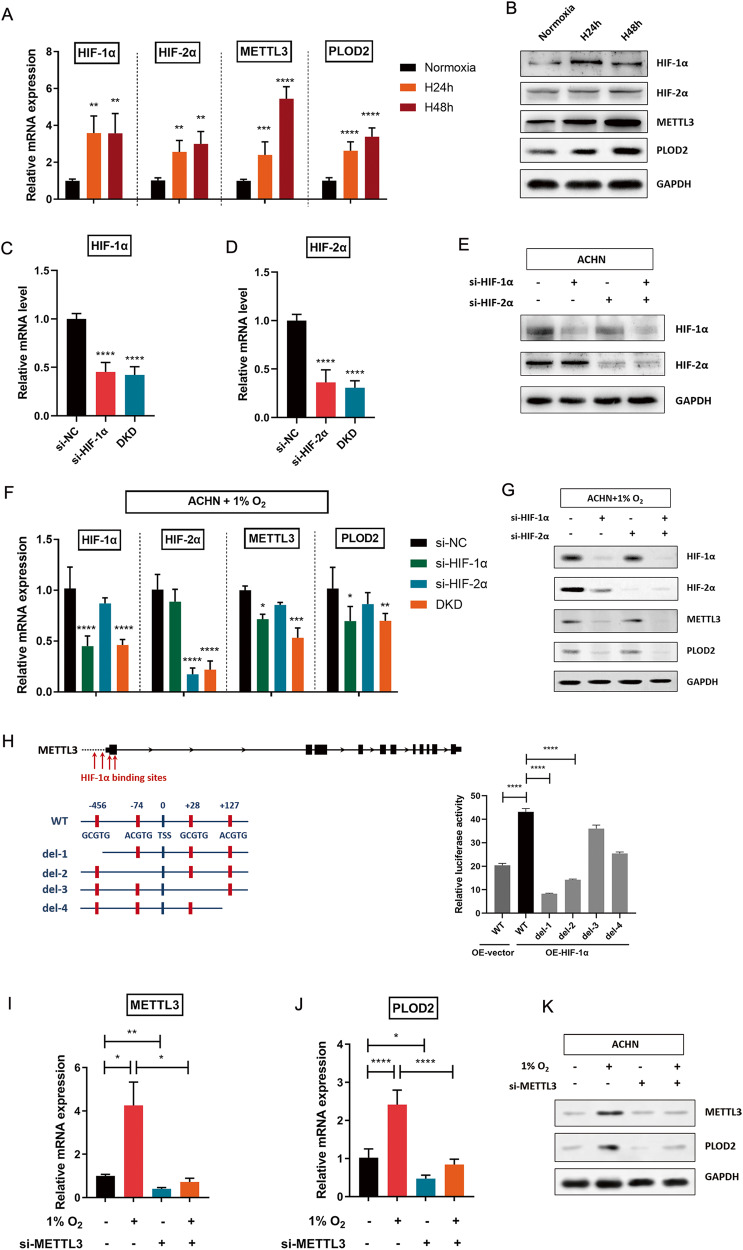
HIFs and METTL3 are required for hypoxia-induced PLOD2 expression. **A**, **B** Expression levels of HIF-1α, HIF-2α, METTL3, and PLOD2 were determined by RT-qPCR (**A**) and western blotting (**B**) after ACHN cells were exposed to normoxia (20% O_2_) or hypoxia (1% O_2_). **P = 0.0013, 0.0022, 0.0087, and 0.0026 (left to right), ***P = 0.0002, ****P < 0.0001. **C**–**E** Examination of the knockdown efficiency when si-HIF-1α, si-HIF-1α, or double knockdown (DKD) were transfected into ACHN cells. ****P < 0.0001. **F**–**G** Silencing of HIFs decreased METTL3 and PLOD2 expression at mRNA level (**F**) and protein level (**G**) in RCC cells under prolonged hypoxia. ****P < 0.0001, *P = 0.0454 and 0.0124 (left to right), ***P = 0.0004, **P = 0.0082. (H) Luciferase reporter assay was used to determine the potential HIF-1α binding sites in METTL3 promoter region. ****P < 0.0001. **I**–**K** Expression levels of METTL3 and PLOD2 were examined in ACHN cells by RT-qPCR (**I**, **J**) and western blotting (**K**) when prolonged hypoxia was combined with METTL3 silencing. **J** *P = 0.0336 (left), 0.0274 (right), **P = 0.0010. K, *P = 0.0323, ****P < 0.0001. Data were represented as mean ± S.D. of three independent experiments.

Hypoxic microenvironment is a common feature in advanced tumors. Hypoxia-inducible factors (HIFs) are key transcriptional activators upon hypoxic stimulation. To discover the potential regulation of hypoxic microenvironment on METTL3 and PLOD2 expression, ACHN cells with moderate endogenous METTL3 expression level were exposed to normoxia (20% O_2_) or hypoxia (1% O_2_). Expression levels of HIFs, METTL3 and PLOD2 were significantly induced under prolonged hypoxia, as determined in both RNA and protein levels (Fig. 4A, B and Supplementary Fig. 2A). To determine whether hypoxia-induced METTL3 and PLOD2 expression were dependent on HIF-1α, HIF-2α, or both, small interfering RNAs (siRNAs) targeting HIF-1α or HIF-2α were designed and transfected into cells. The knockdown efficiency was verified (Fig. 4C–E and Supplementary Fig. 2B). In contrast to the negative control group (si-NC), silencing of HIF-1α (si-HIF-1α) or both HIFs (double knockdown, DKD) abrogated the induction of METTL3 and PLOD2 in RCC cells under prolonged hypoxia (Fig. 4F). Western blotting assay also confirmed that METTL3 and PLOD2 protein levels were increased by hypoxia in normal cells (Fig. 4B), but not in HIF-knockdown cells (Fig. 4G and Supplementary Fig. 2C). Thus, hypoxia induces METTL3 and PLOD2 expression in RCC cells in a HIF-dependent manner. In clinical RCC samples, IHC staining and RT-qPCR evaluation for HIF-1α, METTL3 and PLOD2 expression also addressed positive correlations among these molecules (Supplementary Fig. 3).

To verify the transcriptional regulation of METTL3, we found four potential hypoxia-responsive elements (HREs) in the promoter region of METTL3 containing the HIF-1α-binding consensus sequence 5′-A/GCGTG-3′ (Fig. 4H). Subsequently, dual-luciferase reporter vectors containing wild-type or deletion mutants of METTL3 promoter regions were generated (Fig. 4H). Significant induction of relative luciferase signal was observed in the wild-type form of the METTL3 promoter when co-transfected with HIF-1α-overexpression vectors. However, deletion of sites 1 or 2 resulted in a dramatic decrease of luciferase signal, indicating that both sites located upstream of the transcription start site (TSS) of METTL3 were the functional elements recognized by HIF-1α (Fig. 4H).

The regulatory effect of METTL3 on PLOD2 expression under prolonged hypoxia was further investigated. METTL3 depletion dramatically impaired the hypoxic induction of PLOD2 expression in RCC cells, at both RNA and protein levels (Fig. 4I–K and Supplementary Fig. 2D–E). Furthermore, potent inhibition of PLOD2 expression was observed in METTL3-knockdown cells under normoxic conditions (Fig. 4J, K and Supplementary Fig. 2E). It suggests that there is significant METTL3 activity even under non-hypoxic conditions, which is further increased by prolonged exposure to hypoxia. Taken together, these findings reveal that METTL3 mediates PLOD2 expression, which is dependent on HIF-induction in hypoxic RCC cells.

### METTL3 facilitates PLOD2 expression by enhanced m^6^A-methylation

**Fig. 5:**
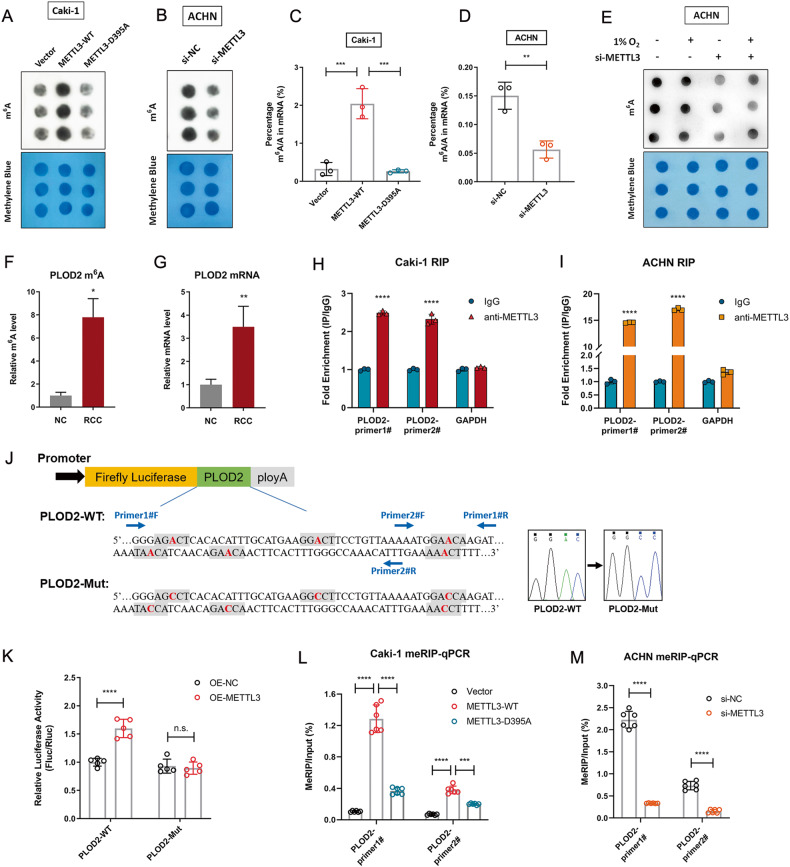
METTL3 mediates m^6^A RNA methylation on PLOD2 mRNA. **A** m^6^A dot-blot assay was used to detect the m^6^A levels in Caki-1 cells with or without METTL3 overexpression. Methylene blue staining was served as loading control. **B** m^6^A dot-blot assay was used to detect the m^6^A levels in ACHN cells with or without silencing METTL3. **C** The m^6^A contained in total RNA was measured by colorimetric quantification in Caki-1 cells with or without over-expressing METTL3. ***P = 0.0004 (left), 0.0003 (right). **D** The m^6^A contained in total RNA was measured by colorimetric quantification in ACHN cells with or without silencing METTL3. **P = 0.0044. **E** ACHN cells with or without METTL3 knockdown were exposed to 20% or 1% O_2_ for 48 h. Total RNA was extracted, and m^6^A levels were determined by a m^6^A dot-blot assay. **F** m^6^A enrichment of PLOD2 mRNA in RCC tissues and matched adjacent normal tissues was measured by meRIP-RT-qPCR. *P = 0.0143. **G** PLOD2 mRNA level in RCC tissues and adjacent normal tissues was detected by RT-qPCR. **P = 0.0019. **H**, **I** RIP analyses of Caki-1 cells (**H**) and ACHN cells (**I**) were performed with anti-METTL3 or IgG antibody followed by qPCR analyses with primers against PLOD2 mRNA. Data represent means ± S.D. of triplicate samples. ****P < 0.0001. **J** Schematic representation of luciferase reporter vectors. **K** Luciferase reporter vectors containing wild-type or mutated m^6^A sites in PLOD2-3’UTR were transfected into 293T cells with or without METTL3 over-expression. Relative luciferase activity (Fluc normalized to Rluc) was measured. ****P < 0.0001, n.s., no significance. **L** MeRIP-RT-qPCR analysis of m^6^A enrichment in the PLOD2 locus in Caki-1 cells with or without METTL3 overexpression. ****P < 0.0001, ***P = 0.0009. **M** MeRIP-RT-qPCR analysis of m^6^A enrichment in the PLOD2 locus in ACHN cells with or without silencing METTL3. ****P < 0.0001. Data were represented as mean ± S.D. of three independent experiments.

To elucidate the molecular mechanisms underlying METTL3-promoted RCC progression, we investigated the role of METTL3 in regulating m^6^A RNA methylation in RCC cells. Enhanced or decreased total m^6^A abundance was associated with METTL3-overexpression or depletion in RCC cells, as determined by RNA m^6^A dot-blot assay (Fig. 5A, B). However, elevated m^6^A methylation was abrogated when METTL3-D395A vector was used (Fig. 5A). Similar results were obtained when m^6^A RNA modifications were colorimetrically quantified in total cellular RNA pools by a m^6^A RNA methylation assay kit (Fig. 5C, D). Next, the effect of hypoxia exposure and alterations in METTL3 expression on m^6^A methylation was investigated. We observed that the increased total m^6^A level induced by prolonged hypoxia was abolished in METTL3-knockdown cells (Fig. 5E). This suggested that the augmented m^6^A RNA modification observed in hypoxic cells is partially attributed to the increased expression of METTL3.

According to our previous m^6^A sequencing data, PLOD2 is one of the most significant elevated hyper-methylated m^6^A transcripts in RCC tissue samples, with a 14.5-fold increase in relative m^6^A enrichment compared with normal controls. Meanwhile, PLOD2 was one of the most highly upregulated genes in RCC tissues [9]. The above results were further confirmed in our RCC cohort (Fig. 5F, G). RIP-RT-qPCR analysis showed that PLOD2 transcripts were significantly enriched by anti-METTL3 antibody precipitation compared with the IgG pull-down group in RCC cell lines (Fig. 5H, I), confirming the direct interaction between METTL3 and PLOD2 mRNA.

Our previous MeRIP-seq data has identified several m^6^A peaks downstream of the last exon of PLOD2 mRNA that match with the RRACU m^6^A consensus sequence [9]. Several m^6^A sites in 3’UTR of PLOD2 mRNA were further predicted by the online m^6^A sites prediction tool SRAMP (http://www.cuilab.cn/sramp) with very high confidence (Fig. 5J). To determine the effect of METTL3-dependent m^6^A regulation on PLOD2 expression, dual-luciferase reporter vectors containing the WT or mutated m^6^A sites of PLOD2-3’UTR fragments were constructed. For the PLOD2-Mut reporter that resists m^6^A modification, adenine (A) to cytosine (C) substitutions (shown in red) were made within m^6^A consensus sequence (Fig. 5J). Luciferase assay showed that METTL3 overexpression largely increased the relative luciferase activity in PLOD2-WT group, but not in PLOD2-Mut group (Fig. 5K). To confirm that METTL3 catalyzes the m^6^A modification of PLOD2, total cellular RNA was immunoprecipitated with an anti-m^6^A specific antibody followed by RT-qPCR (MeRIP-RT-qPCR). In line with previous reports [9, 16], the m^6^A signal was enriched around the stop codon and 3’UTR region of PLOD2 mRNA, as determined by two different pairs of qPCR primers (Fig. 5J, L, M). Moreover, the m^6^A level of PLOD2 mRNA experienced a significant increase after overexpression of METTL3-WT but not METTL3-D395A (Fig. 5L). Conversely, m^6^A level in PLOD2 mRNA was notably decreased upon METTL3 depletion (Fig. 5M).

Taken together, these results strongly suggest that METTL3 binds to PLOD2 mRNA and positively modulates its expression by enhancing m^6^A levels. M^6^A modifications on PLOD2 mRNA can be subsequently recognized by YTHDF1, an m^6^A reader, enhancing the translation of PLOD2 protein [16]. Enhanced PLOD2 expression activates key molecules in signalling pathways related to migration and invasion, thus promoting RCC development [11].

### Targeting of METTL3 abrogates RCC progression in vitro and in vivo

**Fig. 6:**
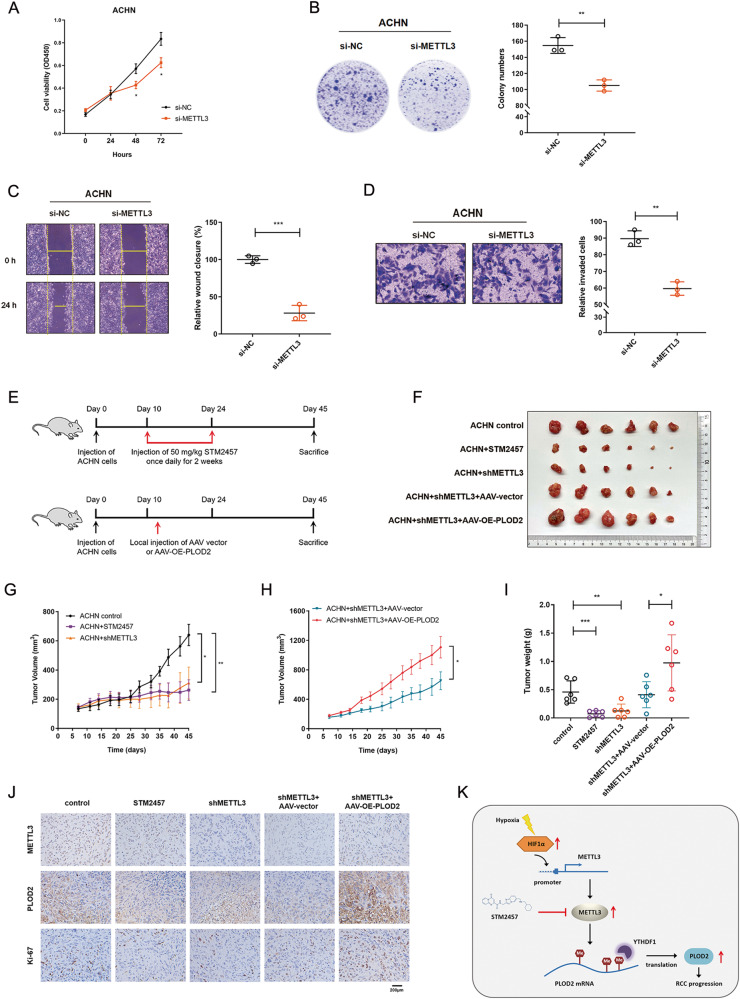
Targeting METTL3 abrogates RCC progression in vitro and in vivo. **A** Cell growth curve of ACHN cells transfected with si-NC or si-METTL3. *P = 0.0441 (left), 0.0328 (right). **B** The proliferation ability after depletion METTL3 in ACHN cells was evaluated by colony formation assay (representative wells were presented). **P = 0.0020. **C** Wound-healing assay was applied for the cell migration in METTL3 knockdown ACHN cells compared with normal controls. ***P = 0.0004. **D** Cell invasion ability after depletion METTL3 in ACHN cells was measured via the transwell cell invasion assay. **P = 0.0011. **E** Schematic diagram illustrates the design of animal experiments. **F** Xenograft tumors derived from ACHN cells with METTL3 inhibitor, METTL3 knockdown, PLOD2 overexpression, or negative control were shown (*n* = 6). Mice were sacrificed 45 days post-injection. **G**, **H** Tumor growth curves in ACHN xenograft tumors with different treatments. Tumor volume was calculated twice a week. *P = 0.0169, **P = 0.0073 (**G**), *P = 0.0288 (**H**). **I** Tumor weight in ACHN xenograft tumors with different treatments. Data represent the means ± S.D. of 6 mice in each group. ***P = 0.0006, **P = 0.0021, *P = 0.0303. **J** Representative IHC staining of METTL3, PLOD2, and Ki-67 in xenograft tumors with different treatments. Scale bar, 200 μm. **K** A mechanism of RCC progression promoted by METTL3-mediated PLOD2 m^6^A-methylation under prolonged hypoxia. Data were represented as mean ± S.D.

To identify the role of METTL3 in RCC cell proliferation, METTL3 expression was depleted in ACHN cells. Knockdown of METTL3 significantly inhibited cell proliferation (Fig. 6A) and colony formation ability (Fig. 6B). An obvious delay in cell migration rate was also observed in METTL3-depletion cells through wound-healing and transwell invasion assays (Fig. 6C, D). The above results indicated that the knockdown of METTL3 could impair RCC progression in vitro.

To further demonstrate our in vitro results, in vivo subcutaneous implantation mouse models were employed (Fig. 6E). METTL3-stable-knockdown ACHN cells were constructed by lentivirus-mediated short hairpin RNA. Cells were subcutaneously injected into the flank regions of BALB/C nude mice (*n* = 6/group). METTL3 depletion significantly reduced the size (Fig. 6F), volume (Fig. 6G) and weight (Fig. 6H) of xenograft tumors. STM2457, a novel and selective METTL3 inhibitor, was discovered recently with a therapeutic effect in acute myeloid leukemia [20]. Here, we examine the effect of STM2457 on RCC progression. It showed that tumor growth was blunted by STM2457 application (Fig. 6F, G, I). Reduced expression of downstream target PLOD2 and the proliferation marker Ki-67 were observed in METTL3 depletion mice by IHC staining (Fig. 6J). Of note, these results suggest that therapeutically targeting METTL3 can be a promising treatment for RCC in clinical applications. Furthermore, a rescue experiment was conducted by local injection with PLOD2-over-expressing-AAV (AAV-OE-PLOD2) around subcutaneous implantation sites in mice treated with sh-METTL3-ACHN cells (Fig. 6E). It showed that the above inhibitory effects of sh-METTL3 were largely abrogated by re-constituted expression of PLOD2, as determined by tumor size (Fig. 6F), volume (Fig. 6H) and weight (Fig. 6I). It also led to an increasing level of Ki-67 in the xenograft tumor tissues (Fig. 6J).

In conclusion, the findings of our study indicate that METTL3 is responsible for mediating m^6^A RNA methylation on PLOD2 mRNA, thereby increasing the expression level of PLOD2 in RCC cells. This study highlights the critical role of hypoxia-induced upregulation of METTL3 in promoting the development of RCC. Furthermore, targeting METTL3 may serve as a novel pharmaceutical intervention for RCC progression (Fig. 6K).

## Discussion

Abnormal expression of oncogenes or tumor suppressor genes plays a critical role in cancer progression. Gene expression can be regulated from different aspects, including transcriptional and post-transcriptional levels. In this study, we provide in vitro and in vivo evidence to elucidate that METTL3, the key m^6^A RNA methyltransferase, plays an oncogenic role in RCC progression. Elevated METTL3 expression predicts poor prognosis in RCC patients. Mechanistically, elevated transcription activity driven by the hypoxic tumor microenvironment is responsible for the aberrant expression of METTL3. METTL3 promotes PLOD2 expression in an m^6^A-dependent manner, thereby facilitating RCC development. Our results demonstrate the importance of the HIF/METTL3/PLOD2 axis in RCC progression.

PLOD2 has been identified with an oncogenic role and acts as a prognostic biomarker in several types of cancers [12, 13]. Emerging evidence indicates that upregulation of PLOD2 affects ECM fiber arrangement, cell adherent ability and directional migration ability in several kinds of cancers, including RCC [14, 16]. In addition, pharmacologic inhibitors of PLOD2, such as minoxidil has been confirmed to have an anti-metastasis effect in vitro and in vivo [12, 21]. However, the regulatory mechanisms of PLOD2 at the post-transcriptional level are still elusive. In a recent study, we reported that PLOD2 expression and the m^6^A enrichment on its transcript were dramatically elevated in RCC samples [9]. Consistent with our findings, a recent study by Cao et al. showed that the expression level and m^6^A methylation level of PLOD2 were significantly increased both in RCC tissues and RCC cell lines [16]. These results suggested that m^6^A RNA modifications were involved in PLOD2 expression regulation. In this study, we focused on METTL3, the major component of the m^6^A methyltransferase complex. The specific m^6^A modification sites in PLOD2 mRNA were identified and confirmed. Enhanced expression of METTL3 in RCC catalyzes the m^6^A modifications at 3’UTR region of PLOD2 transcripts, thus promoting PLOD2 expression.

It is well-established that m^6^A methylation has a modulatory effect on RNA stability, which is mediated by two major families of m^6^A “readers”: the YTH family and the IGF2BP family [22, 23]. Among these, the YTHDF1 protein plays a key role in promoting the translation of m^6^A-modified mRNAs [24, 25]. Previous studies have demonstrated that YTHDF1 is involved in the m^6^A-regulated expression of PLOD2. Specifically, YTHDF1 was found to interact with PLOD2 transcripts that are enriched with m^6^A modifications in RCC cells, thereby promoting the translation of PLOD2 protein [16]. Increased PLOD2 expression is crucial for tumor invasion and metastasis. On the one hand, PLOD2 induces PI3K/AKT signaling [26, 27], thereby promoting the expression of downstream EMT-related molecules and the formation of focal adhesion [28, 29]. On the other hand, PLOD2 may contribute to cancer progression by modulating aberrant collagen cross-link and maturation [30]. A recent study reported that matrix-metalloprotease-cleaved type I collagen activates discoidin domain receptor 1 (DDR1)-NF-κB-p62-NRF2 signaling to promote tumor growth [31]. Thus, it is worth investigating if PLOD2 affects RCC prognosis by altering DDR1 signaling and type I collagen deposition.

Aberrant expression of m^6^A methyltransferase METTL3 has been reported with oncogenic role in various cancer types, including pancreatic cancer [32], bladder cancer [33], liver cancer [34], and acute myeloid leukemia [35]. METTL3 has been reported to correlate with malignant progression and have potential predictive value in RCC [36, 37]. In the present study, we discover that METTL3 promotes RCC progression in vitro and in vivo. Mechanistically, METTL3 induces expression of tumor-promoting gene PLOD2 dependent on its methylase activity. METTL3 mediates m^6^A RNA methylation and eventually promotes the translation of the PLOD2 protein. Therefore, the oncogenic role of METTL3 in RCC is mediated, at least partially, by elevated PLOD2 expression. In addition, highly expressed METTL3 is observed in advanced RCC patients in our study, and is correlated with a poor survival rate. This result was consistent with previous reports [36, 37]. The mechanisms for the dysregulation of METTL3 was further investigated in the present study. Hypoxia-inducible factor HIF-1α was confirmed as upstream regulator of METTL3 expression with defined binding sites. This result enriched the understanding of METTL3 expression regulation at the transcriptional level.

Oxygen deprivation is a common feature of advanced solid tumors due to the rapid growth of tumor cells and the insufficient oxygen supply from adjacent blood vessels. Intratumoral hypoxia can trigger a variety of cellular responses, including angiogenesis, apoptosis, metastasis, drug resistance, and immunosuppression [38–40]. Cellular responses to hypoxic microenvironments are mainly mediated by hypoxia-inducible factors (HIF-1 and -2), which can activate the transcription of specific downstream target genes. Previous studies have shown that PLOD2 expression can be induced by hypoxia thus affecting cell morphology, adherent ability, tumor migration and invasion ability [14, 15]. In this study, we further demonstrat that intracellular hypoxia is the driving force and act as an upstream regulator of METTL3 and PLOD2 expression. As the initial signal, hypoxia induces the upregulation of HIF-1α, which subsequently recognizes and binds to METTL3 promoter region to enhance METTL3 transcription. METTL3 then mediates m^6^A RNA methylation on PLOD2 mRNA, and eventually promotes the expression of PLOD2 protein and RCC progression. Another study conducted in hepatocellular carcinoma cells reported that METTL3 also had a binding relationship with HIF-1α mRNA to promote the m^6^A modification and expression of HIF-1α [41]. Therefore, METTL3 and HIF-1α form a positive feedback loop in regulating each other, which is further confirmed by the latest research results of two other independent laboratories [42, 43].

This study addressed the central position of METTL3 in the HIF/METTL3/PLOD2 axis of advanced RCC. Thus, targeting METTL3 by specific inhibitors highlights the importance of developing effective therapies under hypoxic conditions. STM2457 is a highly potent and selective METTL3 inhibitor discovered recently with therapeutic effect in acute myeloid leukemia [20]. It also showed anti-tumor effect in cell models of intrahepatic cholangiocarcinoma by inhibiting cell proliferation, invasion and migration [7]. Our in vivo experiments revealed that the administration of STM2457 significantly abrogated RCC progression by reducing xenograft tumor growth, suggesting that METTL3 may serve as a potential target for RCC therapy.

In conclusion, we propose in this study a novel regulatory mechanism in which METTL3 promotes the expression of the oncogene PLOD2 in m^6^A-dependent way. It provides novel insights into the molecular mechanisms of METTL3-induced tumor progression in RCC under prolonged hypoxia and identifies potential therapeutic targets specific to advanced RCC.

## Materials and methods

### Patient samples

A total of 39 RCC tissues and tumor-adjacent normal tissues were collected from RCC patients at the time of surgery. All RCC patients in this study were recruited from the Department of Urology, The Third Affiliated Hospital of Soochow University, China. Tissue samples were immediately separated into 1.5 ml RNase-free centrifuge tubes and stored at −80 °C until RNA isolation or protein extraction. This study was approved by the Ethics Committee of The Third Affiliated Hospital of Soochow University, and written informed consent was obtained from all participants. All procedures performed in studies involving human participants were compliant with ethical standards. The demographics and clinical characteristics of participants are listed in Supplementary Table S2.

### Cell lines and cell culture

The human normal renal epithelial cell line HK-2 and RCC cell lines Caki-1, ACHN, 786-o, and 769-p were purchased from the National Collection of Authenticated Cell Cultures (Shanghai, China). Caki-1 cells were cultured in McCoy’s 5A medium (Gibco, USA) according to the product manual. HK-2, ACHN, 786-o, and 769-p cell lines were cultured in RPMI-1640 medium (Gibco, USA), which were all supplemented with 10% fetal bovine serum (Invitrogen, USA) and 1% penicillin-streptomycin (Gibco, USA) at 37 °C under a humidified atmosphere of 5% CO_2_. For hypoxia exposure, cells were placed in a modular incubator chamber (New Brunswick™ Galaxy® 48R, Eppendorf, Germany), which was flushed with gas mixture containing 1% O_2_, 5% CO_2_, and 94% N_2_ at 37 °C.

### Cell transfection and stable cell lines establishment

For transient modulating gene expression in cell lines, short interfering RNA (siRNA) sequences for HIF-1α, HIF-2α, and METTL3 were directly synthesized (RiboBio, Guangzhou, China), with nonspecific siRNAs as a negative control. To upregulate METTL3, wild-type (WT) or mutant (D395A, K211R) cDNA fragments were amplified and inserted into pcDNA3.1 vectors. Lipofectamine 3000 reagent (Invitrogen, USA) was used for transfection according to the manufacturer’s instructions.

For stable knockdown of METTL3, lentiviral vectors (hU6-MCS-CBh-gcGFP-IRES-puromycinshRNA) encoding a short hairpin RNA (shRNA) targeting METTL3 or scrambled shRNA (sh-NC) were applied (GeneChem, Shanghai, China). Stably infected cells were screened with 2.5 μg/mL puromycin (Selleck, Shanghai, China) treatment for two weeks. The stably expressing cell lines were confirmed using RT-qPCR and western blotting assays. Sequences of shRNAs and siRNAs used in this study are listed in Supplementary Table S1.

### TMA and IHC staining

TMA was constructed from 90 pairs of formalin-fixed, paraffin-embedded renal cancer tissues and matched normal tissues (Tufei Biotech, Shanghai, China). Demographics and clinical characteristics of these samples are listed in Supplementary Table S2. After antigen retrieval, TMAs were blocked and stained with anti-METTL3 antibody (ab195352, abcam, UK). The degree of immunostaining was evaluated by two independent pathologists. IHC score was calculated as staining intensity (0 = negative, 1 = weak staining, 2 = moderate staining, 3 = strong staining, 4 = severe staining) multiplying staining area (0 = 0%, 1 = 1–25%, 2 = 26–50%, 3 = 51–75%, 4 = above 75%).

For IHC staining, tissues were fixed in 10% (v/v) formaldehyde in PBS, embedded in paraffin, and cut into 4 μm sections. Slides were incubated with specific primary antibodies against METTL3 (1:50, ab195352, Abcam, UK), PLOD2 (1:500, 21214-1-AP, Proteintech, USA), HIF-1α (1:100, ab51608, Abcam, UK) and Ki-67 (1:400, ab16667, Abcam, UK) respectively, at 4 °C overnight. Thereafter, secondary antibodies were added followed by DAB staining and haematoxylin counter-staining.

### Western blot assay

Cells or tissue samples were lysed in RIPA buffer (Beyotime, Shanghai, China) mixed with protein loading buffer (Solarbio, Beijing, China). Extracted protein were loaded onto SDS-polyacrylamide gels and fully electrophoresed. After separation, proteins were transferred to PVDF membranes. Membranes were incubated with specific primary antibodies (at 1:1000 dilution) and HRP-conjugated secondary antibodies (at 1:10000 dilution). The protein level was detected with an enhanced chemiluminescence system (Tanon, Shanghai, China). The following primary antibodies were used: anti-METTL3 (ab195352, abcam, UK), anti-PLOD2 (21214-1-AP, proteintch, USA), anti-HIF-1α (ab179483, abcam, UK), anti-HIF-2α (ab8365, abcam, UK), anti-GAPDH (ab011, Multi Sciences, Hangzhou, China). All of the experiments were performed at least three times, and the most representative results were shown. The original western blots can be found in Supplemental Material.

### RNA isolation and quantitative RT-PCR (RT-qPCR)

Total RNA was extracted using TRIzol reagent (Invitrogen, USA) according to the manufacturer’s instructions. RNA was reverse-transcribed into cDNA with PrimeScript™ RT reagent Kit (Takara Biomedical Technology, Beijing, China). SYBR Green-based RT-qPCR was performed using TB Green™ Premix Ex Taq (Takara Biomedical Technology, Beijing, China) in Applied Biosystems™ *7500* real-time PCR system (Thermo Fisher Scientific, USA). The expression of the target genes was calculated by the 2^−ΔΔCt^ method, with the levels normalized to internal control GAPDH. The primer sequences are listed in Supplementary Table S1.

### Cell proliferation assay

Caki-1 and ACHN cells suspended in McCoy’s 5A and RPMI-1640 medium were seeded into 96-well dish at a density of 2000 cells per well. Twenty-four hours after transfecting siRNAs or vectors, the viability of Caki-1 and ACHN cells was determined by Cell Counting Kit 8 (Solarbio, Beijing, China) according to the manufacturer’s instructions. Cell viability was measured at OD 450 nm with the Bio Tek Gen5 system (BioTeck, USA).

### Colony formation assay

Cells were seeded into six-well dish at a density of 1000 cells per well and were transfected with siRNAs or vectors to knockdown or overexpression METTL3 after cell attachment. After two weeks, cells were fixed with 4% formaldehyde followed by crystal violet staining. Colonies were photographed and counted using Image J software (NIH, USA).

### Wound-healing and transwell assay

Transfected Caki-1 and ACHN cells were seeded in six-well plates to conduct a wound-healing assay. When cells were grown to 100% confluence, scratches were performed in the middle of the plates using micropipette tips. Photographs were taken at the time points 0 h and 24 h.

Invasion ability was measured by transwell assay. The bottom of the transwell chambers was covered with Matrigel (Corning, USA), and the surfaces of the Matrigel were seeded with Caki-1 and ACHN cells that were suspended in 200 µl serum-free medium. Chambers were placed into 24-well plate with 800 µl culture medium supplemented with 10% FBS. After incubation for 24 h at 37 °C, cells on the bottom of transwell chambers were fixed with 4% formaldehyde and then stained with crystal violet.

### Cell cycle assay

Caki-1 and ACHN cells were transfected with siRNAs or vectors to manipulate METTL3 expression before collection. Cell cycle analysis was carried out using the cell cycle staining kit (#BB4104, BestBIo, Shanghai, China). The data was detected by flow cytometry (BD Biosciences, CA, USA) and analyzed using BD FACSDiva software.

### m^6^A RNA methylation assay

To colorimetrically quantify methylated m^6^A in RNA, 200 ng of RNA isolated from different tissues or cells was added into the 96-well microplate and the m^6^A contained in RNA was measured using the m^6^A RNA Methylation Quantification Kit (Colorimetric, ab185912, abcam, UK) at OD 450.

For RNA m^6^A dot-blot assay, 600 ng RNA was spotted over a membrane paper and cross-linked using ultraviolet rays. Membranes were incubated with specific anti-m^6^A antibody (ab284130, abcam, UK) and secondary antibody after being blocked. Membranes were visualized using chemiluminescence system (Tanon, Shanghai, China). The same amount of RNA was spotted on the membranes, stained with 0.02% methylene blue as loading control.

### MeRIP-RT-qPCR

Total RNA was extracted from Caki-1 and ACHN cells using TRIzol reagent (Invitrogen, USA). RNA was fragmented into 100 nt or smaller fragments using RNA fragmentation reagent in GenSeq^®^ m^6^A MeRIP Kit (GS-ET-001, GenSeq Inc., Shanghai, China) according to the instruction. A total of 18 µg of fragmented RNA for each sample was used for immunoprecipitation, and the rest 1 µg RNA was used as input. Immunoprecipitation enrichment was performed using a monoclonal antibody that recognizes m^6^A RNA modifications. After immunoprecipitation, RNA fragments were eluted from the beads and purified before RT-qPCR. Primer sequences used for MeRIP-RT-qPCR are listed in Supplementary Table S1.

### RNA immunoprecipitation (RIP)

Caki-1 and ACHN cells (5 × 10^7^ for each sample) were harvested and lysed in RIP lysis buffer, which then underwent RIP assays using Magna RIP Kit (Millipore, USA) according to the instruction. After centrifuged at 4 °C, the supernatant was then incubated with anti-METTL3 antibody (ab195352, abcam, UK) and negative control IgG (AS070, ABclonal, Wuhan, China) at room temperature. The immunoprecipitated RNA was purified and detected by RT-qPCR. PCR amplification of GAPDH was used as a negative control. Primer sequences used for RIP-RT-qPCR are listed in Supplementary Table S1.

### Luciferase reporter assay

For promoter reporter assay, the WT or deletion mutants of METTL3 promoter regions were synthesized and cloned into the dual-luciferase vector pGL3-basic (Promega, USA). Dual-luciferase reporter constructs and the internal control vector pRL-TK were transfected at a ratio of 10:1 using Lipofectamine 3000 (Invitrogen, CA).

For m^6^A reporter assay, DNA fragments of PLOD2-3’UTR region containing the WT m^6^A motifs, or mutated m^6^A sites (RRACH to RRCCH) were synthesized and cloned into pmirGlo luciferase reporter vector (Promega, USA). Cells with different treatments were seeded in the wells of a 12-well plate and transfected with a mixture of dual-luciferase reporter constructs and the internal control vector at a ratio of 20:1 using Lipofectamine 3000 reagent (Invitrogen, USA).

Twenty-four hours post-transfection, luciferase activity was measured using the Dual-Luciferase^®^ Reporter Assay System (Promega, USA). Firefly luciferase activity was normalized to renilla luciferase activity for each well.

### Animal experiments

ACHN cells were infected by recombinant lentiviruses to stable knockdown METTL3. To construct the subcutaneous implantation model, 5 × 10^6^ cells were resuspended in 200 μl PBS with matrigel and subcutaneously injected into the flank regions of female BALB/C nude mice (5–6 weeks, *n* = 6/group).

METTL3 inhibitor STM2457 (HY-134836, MCE, USA) was dissolved in 20% (w/v) 2-hydroxyproply β-cyclodextrin vehicle (H107, Sigma, USA). Day 10 after cell injection, STM2457 was delivered to the mice via intraperitoneal injection at a dose of 50 mg/kg once daily for 2 weeks (14 treatments). For rescue experiments, two groups of mice were locally injected with 20 µL (2.50 × 10^13^ viral particles/mL) adeno-associated virus 9 (AAV9) overexpression PLOD2 or AAV9-Control into each tumor when the subcutaneous xenografts reached approximately 200 mm^3^.

The width (*W*) and length (*L*) of the tumors were measured twice a week, and the volume (*V*) of each tumor was calculated using the formula *V* = (*W*^2^ × *L*/2). Mice were euthanized in the seventh week, and xenografts were isolated for further studies. All animal experiments were performed humanely in compliance with guidelines reviewed by the Animal Ethics Committee of The Third Affiliated Hospital of Soochow University.

### Statistical analysis

The GraphPad Prism 9.0 software and SPSS 22.0 software were used for the statistical analysis. Firstly, data were tested through normal distribution. Student’s *t* test was used for comparisons between two groups. Comparisons among multiple groups were applied by one-way analysis of variance (ANOVA) followed by Dunnett’s test. Survival analysis was measured by Kaplan–Meier method, and the differences between groups were evaluated by log-rank test. Correlations between gene expression levels were analyzed by the Pearson correlation coefficient. *P* values < 0.05 were considered to be significant.

### Supplementary information

Supplementary material

Supplementary material-original western blots

reproducibility checklist

## Data Availability

All data generated or analyzed during this study are included in this published article and its supplementary information files.
