# Supplementary material for: METTL3 facilitates renal cell carcinoma progression by PLOD2 m6A-methylation under prolonged hypoxia
Source: Cell Death Dis. 2024 Jan 17;15(1):62. doi: 10.1038/s41419-023-06411-w (PMC10794171; doi:10.1038/s41419-023-06411-w)
Supplement: Supplementary file 1 — Supplementary material [file 41419_2023_6411_MOESM1_ESM.doc]

**Supplementary material**

**Supplementary Table S1. Sequences of primers, shRNAs and siRNAs.**

**Supplementary Table S2. Demographics and clinical characteristics of participants.**

**Supplementary Figure 1. The role of METTL3 on PLOD2 expression induction.**

**Supplementary Figure 2. Statistical analysis of western blotting results displayed in Figure 4.**

**Supplementary Figure 3. HIF1α, METTL3 and PLOD2 expression in clinical samples.**

**Supplementary Table S1. Sequences of primers, shRNAs and siRNAs.**

| **Target** | **Sequence (5'-3')** |
| --- | --- |
| METTL3 siRNA-1 | CAAGTATGTTCACTATGAA |
| METTL3 siRNA-2 | GCAAGTATGTTCACTATGA |
| HIF1α siRNA | CCAGCAACTTGAGGAAGTA |
| HIF2α siRNA | GCCGTACTGTCAACCTCAA |
| METTL3 shRNA | CAAGTATGTTCACTATGAA |
| METTL3 qPCR-F | CAAGCTGCACTTCAGACGAA |
| METTL3 qPCR-R | GCTTGGCGTGTGGTCTTT |
| PLOD2 qPCR-F | TGGCATGAACACGTCTTTG |
| PLOD2 qPCR-R | TTGGCCCAAAGTGAAGTTG |
| HIF1α qPCR-F | AGAAACCACCTATGACCTGCT |
| HIF1α qPCR-R | CGACTGAGGAAAGTCTTGCTA |
| HIF2α qPCR-F | AACGAGTCCGAAGCCGAAG |
| HIF2α qPCR-R | GCTCCACCTGTGTAAGTCCCA |
| GAPDH qPCR-F | GGGAAGGTGAAGGTCGGAGT |
| GAPDH qPCR-R | GGGGTCATTGATGGCAACA |
| PLOD2 MeRIP-RT-qPCR-1F | TGGCATGAACACGTCTTTG |
| PLOD2 MeRIP-RT-qPCR-1R | TTGGCCCAAAGTGAAGTTG |
| PLOD2 MeRIP-RT-qPCR-2F | CTGGGAGACTCACACATTTGC |
| PLOD2 MeRIP-RT-qPCR-2R | AGACGTGTTCATGCCAGTCA |
| PLOD2 RIP-RT-qPCR-1F | TGGCATGAACACGTCTTTG |
| PLOD2 RIP-RT-qPCR-1R | TTGGCCCAAAGTGAAGTTG |
| PLOD2 RIP-RT-qPCR-2F | CTGGGAGACTCACACATTTGC |
| PLOD2 RIP-RT-qPCR-2R | AGACGTGTTCATGCCAGTCA |
| GAPDH RIP-RT-qPCR-F | CAAGAAGGTGGTGAAGCAGG |
| GAPDH RIP-RT-qPCR-R | GTCAAAGGTGGAGGAGTGGG |

**Supplementary Table S2. Demographics and clinical characteristics of participants.**

|  | **Tissue microarray**  **(n=90)** | **Validation RCC cohort**  **(n=39)** |
| --- | --- | --- |
| **Age, years** | 59.1 (11.3) | 65.0 (9.9) |
| **Gender, n (%)** |  |  |
| **Male** | 59 (65.6） | 20 (51.3) |
| **Female** | 31 (34.4) | 19 (48.7) |
| **pT stage, n (%)** |  |  |
| **pT1** | 58 (64.4) | 27 (69.2) |
| **pT2** | 26 (28.9) | 4 (10.3) |
| **pT3** | 6 (6.7) | 7 (17.9) |
| **pT4** | 0 (0) | 1 (2.6) |
| **Lymph node metastasis, n (%)** |  |  |
| **pN0** | 88 (97.8) | 37 (94.9) |
| **pN1** | 2 (2.2) | 2 (5.1) |
| **Distant metastasis, n (%)** |  |  |
| **M0** | 88 (97.8) | 38 (97.4) |
| **M1** | 2 (2.2) | 1 (2.6) |
| **TNM stage, n (%)** |  |  |
| **Ⅰ** | 58 (64.4) | 26 (66.7) |
| **Ⅱ** | 24 (26.7) | 5 (12.8) |
| **Ⅲ** | 6 (6.7) | 7 (17.9) |
| **Ⅳ** | 2 (2.2) | 1 (2.6) |


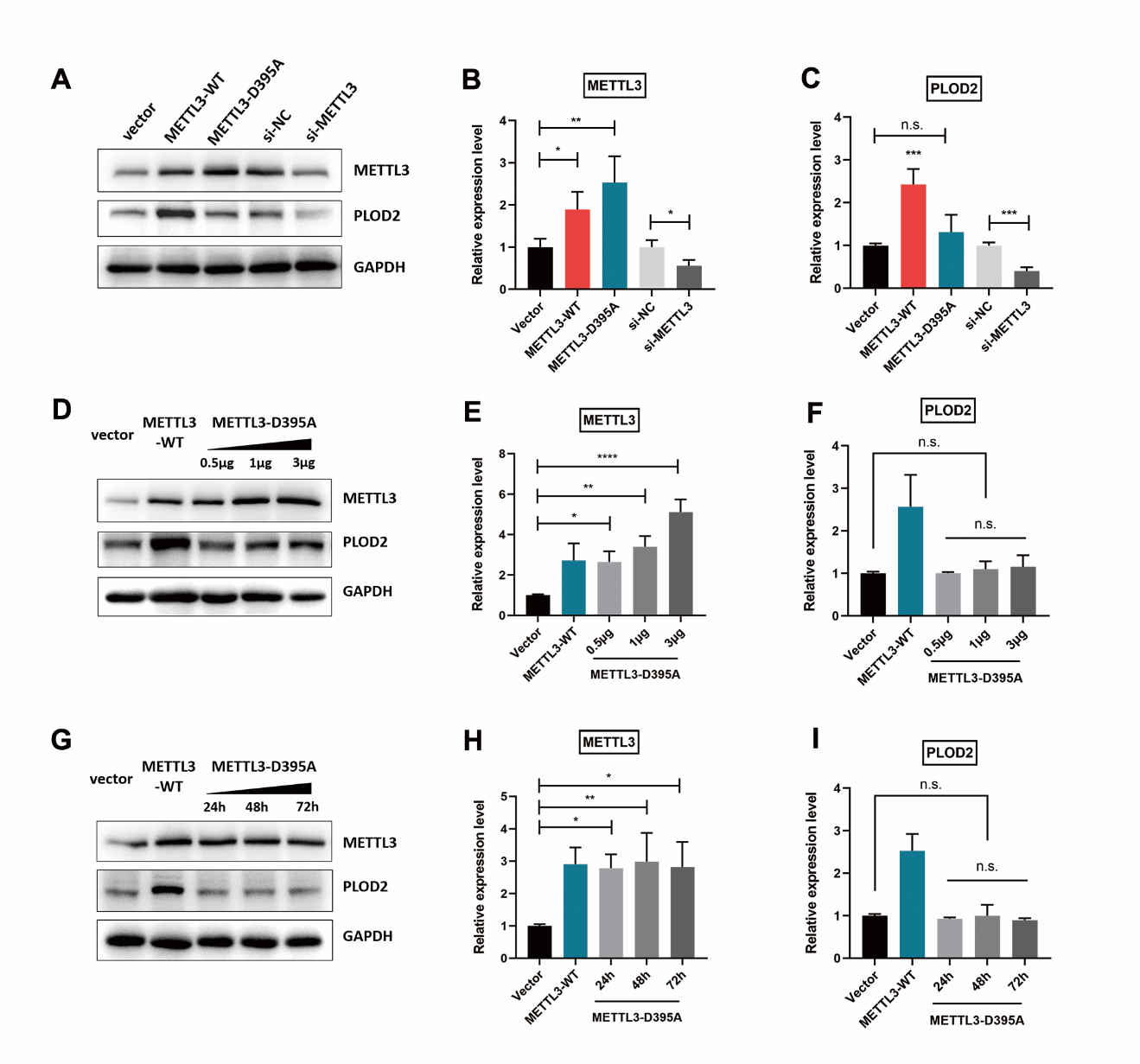


**Supplementary Figure 1. The role of METTL3 on PLOD2 expression induction.**

(A-C) Protein levels of METTL3 and PLOD2 in ACHN cells with METTL3 overexpression or depletion were determined by western blotting. ***P* =0.0011, **P* =0.0270 and 0.0230 (left to right), ****P* =0.0002 and 0.0008 (left to right). (D-F) Protein levels of METTL3 and PLOD2 in Caki-1 cells in response to different dose of METTL3-D395A overexpression. Cells were harvested 24h after transfection. **P* =0.0186, ***P* =0.0016, *****P*<0.0001. (G-I) Protein levels of METTL3 and PLOD2 were assessed in Caki-1 cells at different time points following METTL3-D395A overexpression. Cells were transfected with a dose of 1 μg/well. **P* =0.0161 (left) and 0.0142 (right), ***P* =0.0085, n.s., no significance. Data were represented as mean ± S.D. of three independent experiments.


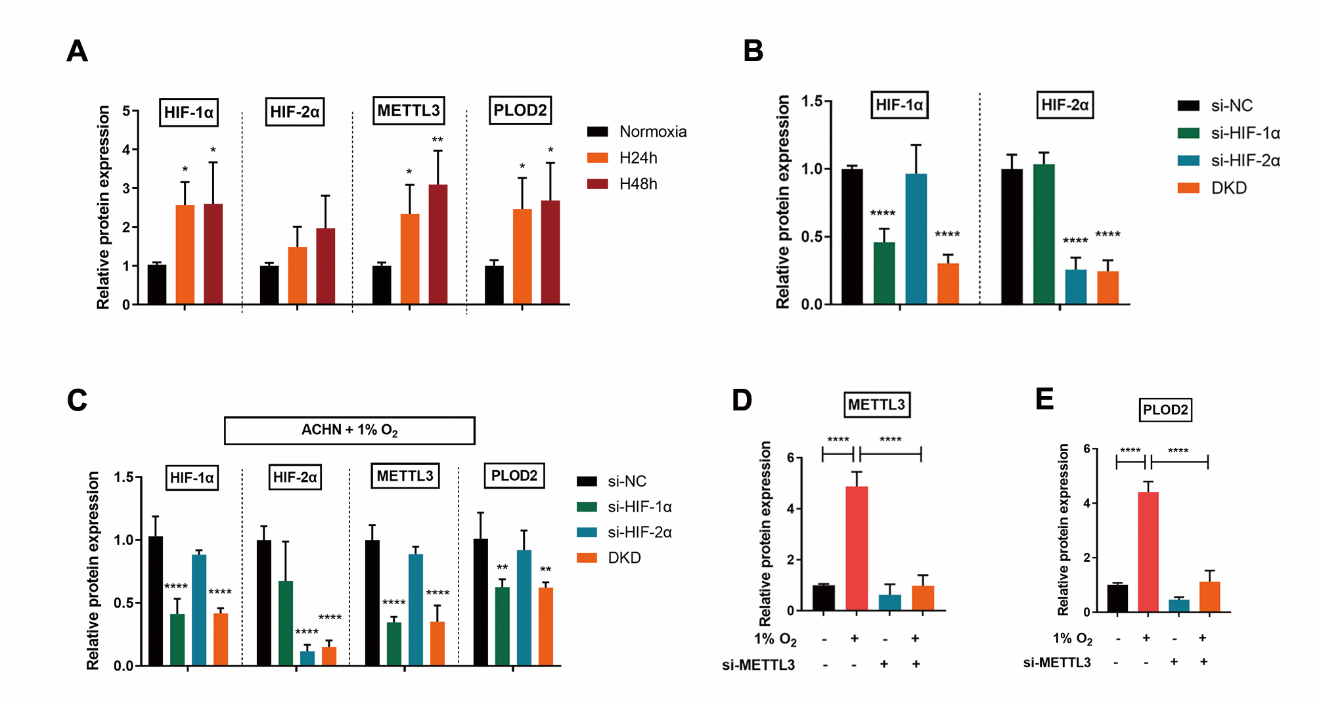


**Supplementary Figure 2. Statistical analysis of western blotting results displayed in Figure 4.**

(A) A statistical analysis of the western blotting results of Fig. 4B after ACHN cells were exposed to normoxia (20% O_2_) or hypoxia (1% O_2_). **P* =0.0352, 0.0208, 0.0417, 0.0239 and 0.0123 (left to right), ***P* =0.0018. (B) A statistical analysis of the western blotting results of Fig. 4E after si-HIF-1α, si-HIF-1α, or double knockdown (DKD) were transfected into ACHN cells. *****P* <0.0001. (C) A statistical analysis of the western blotting results of Fig. 4G after silencing of HIFs in ACHN cells under prolonged hypoxia. ***P* =0.0043 and 0.0040 (left to right), *****P* <0.0001. (D-E) A statistical analysis of the western blotting results of Fig. 4K when prolonged hypoxia was combined with METTL3 silencing in ACHN cells. *****P* <0.0001.


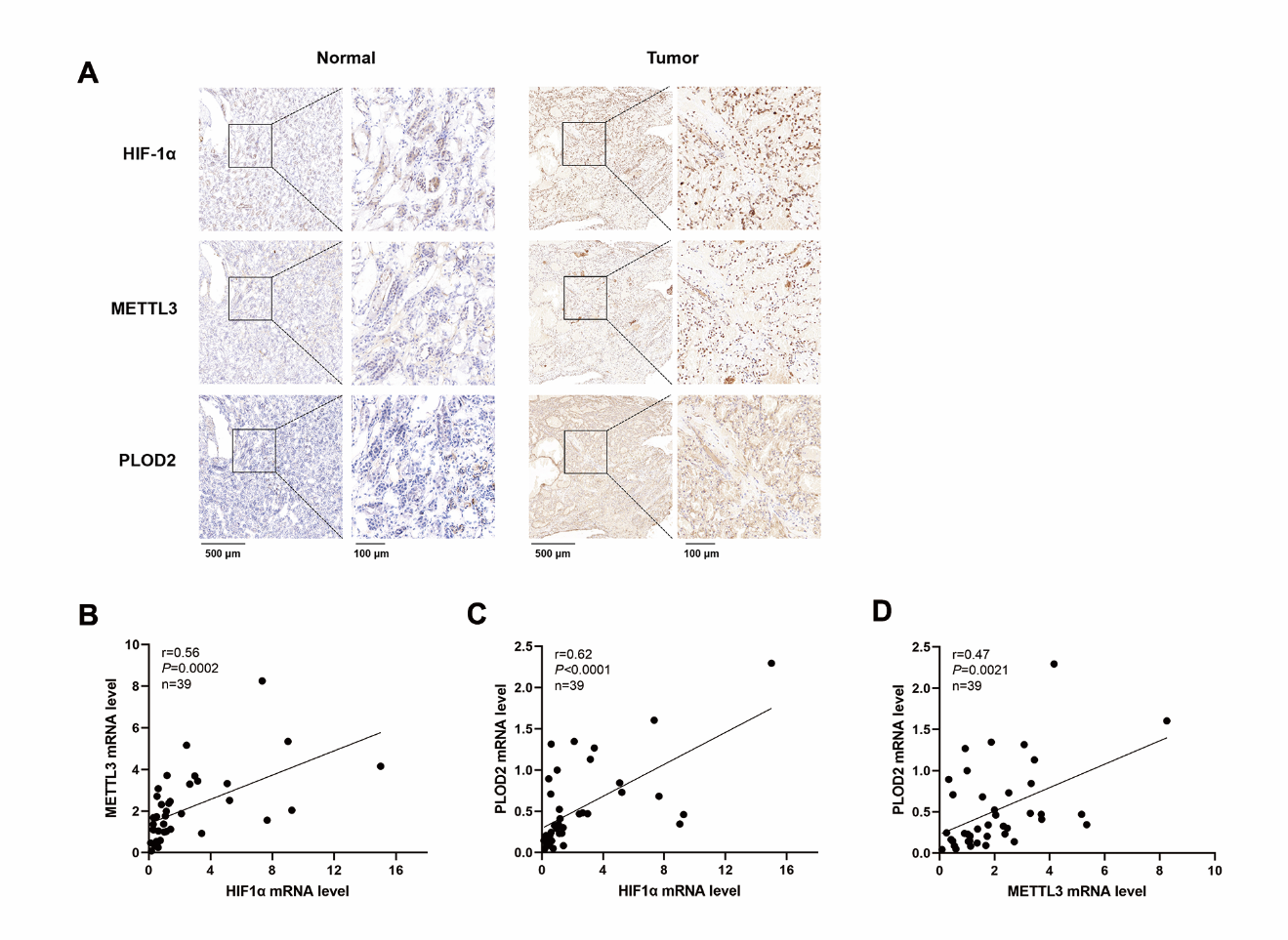


**Supplementary Figure 3. HIF1α, METTL3 and PLOD2 expression in clinical samples.**

(A) HIF1α, METTL3 and PLOD2 expression in the normal renal tissues and tumor area of RCC tissues detected by IHC. (B-D) Positive correlations among the expression levels of HIF1α, METTL3, and PLOD2 in renal tissue samples.
