# Supplementary material for: METTL3 facilitates renal cell carcinoma progression by PLOD2 m6A-methylation under prolonged hypoxia
Source: Cell Death Dis. 2024 Jan 17;15(1):62. doi: 10.1038/s41419-023-06411-w (PMC10794171; doi:10.1038/s41419-023-06411-w)

**Supplementary material**

**Original western blots**

**Figure 1G Increased METTL3 expression in RCC tissues compared to their adjacent normal tissues was verified by western blotting.**


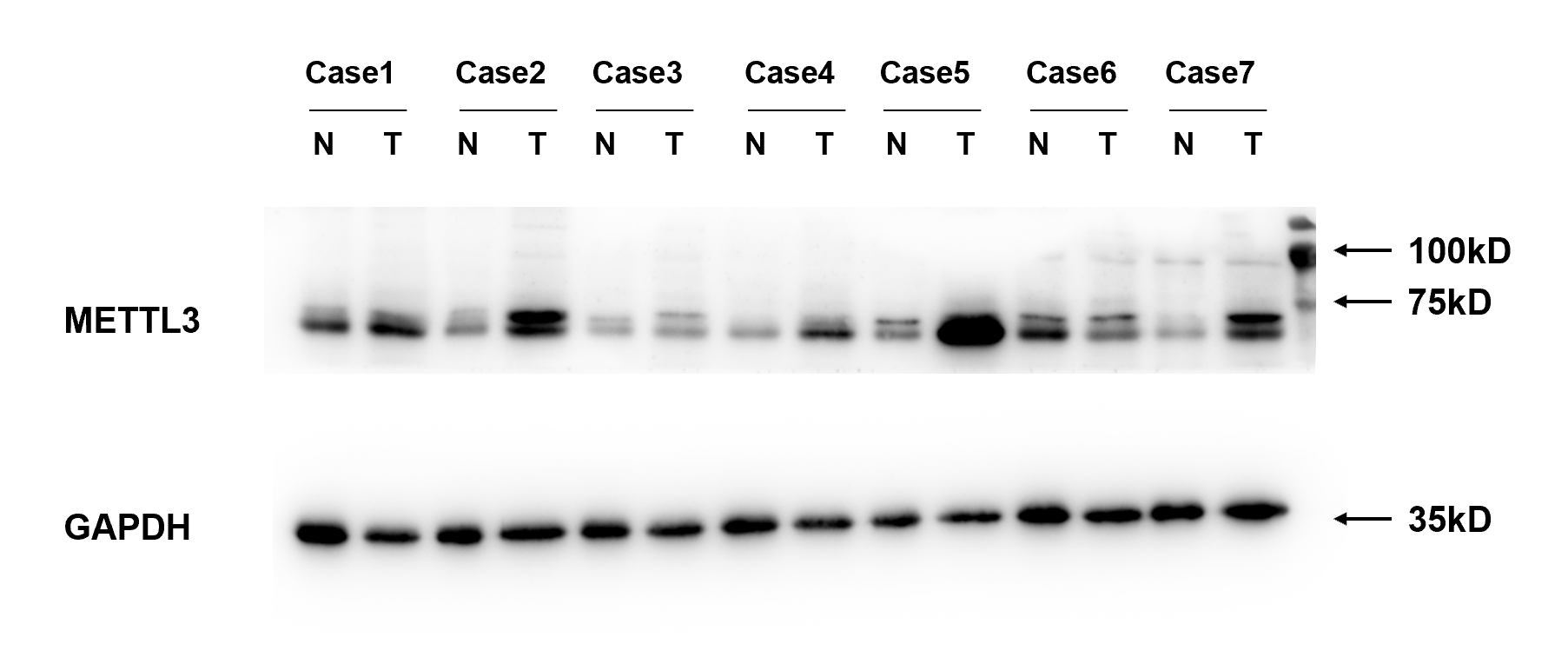


**Figure 2B Western blotting analysis of METTL3 protein levels in four RCC cell lines and normal renal epithelial cells (HK-2).**


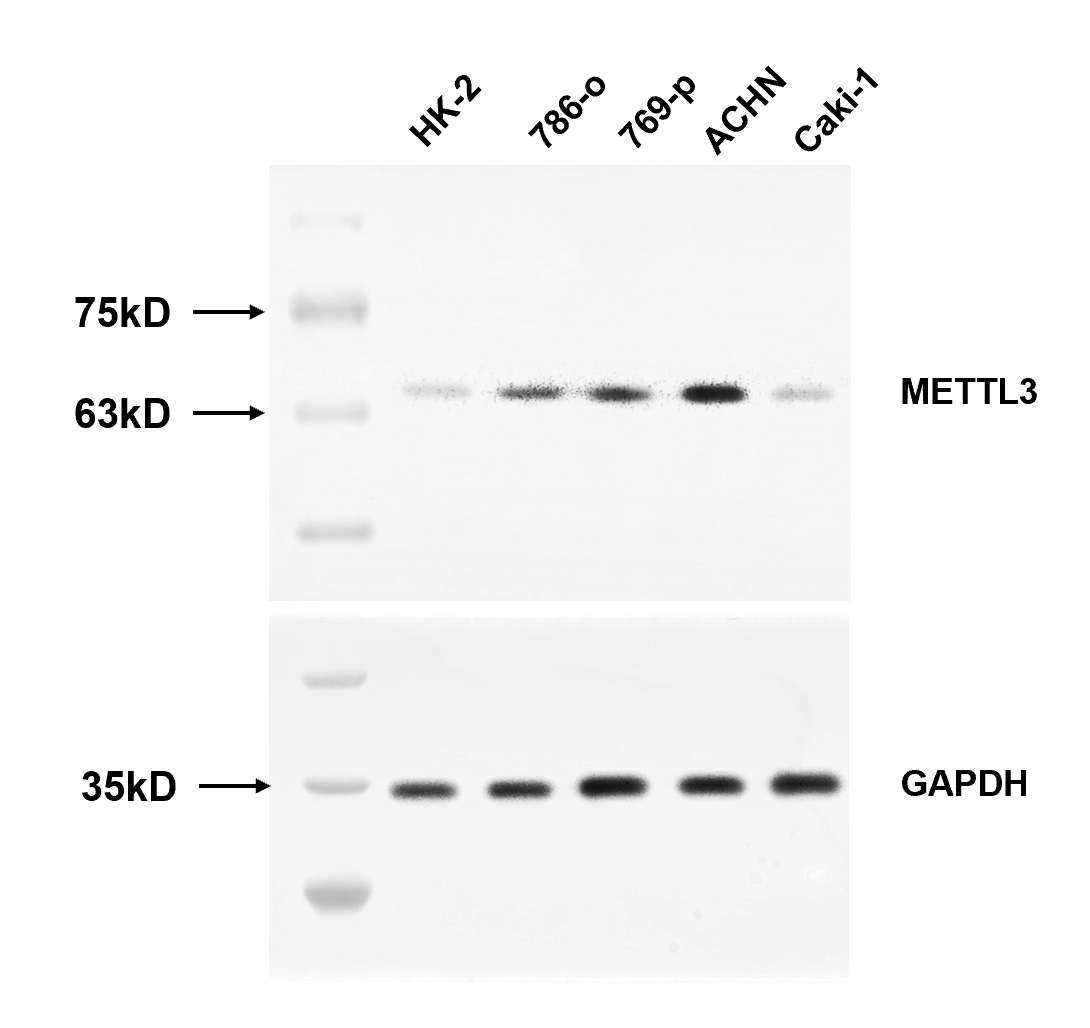


**Figure 2D METTL3 protein levels in Caki-1 cells transfected with METTL3 over-expressing vectors.**


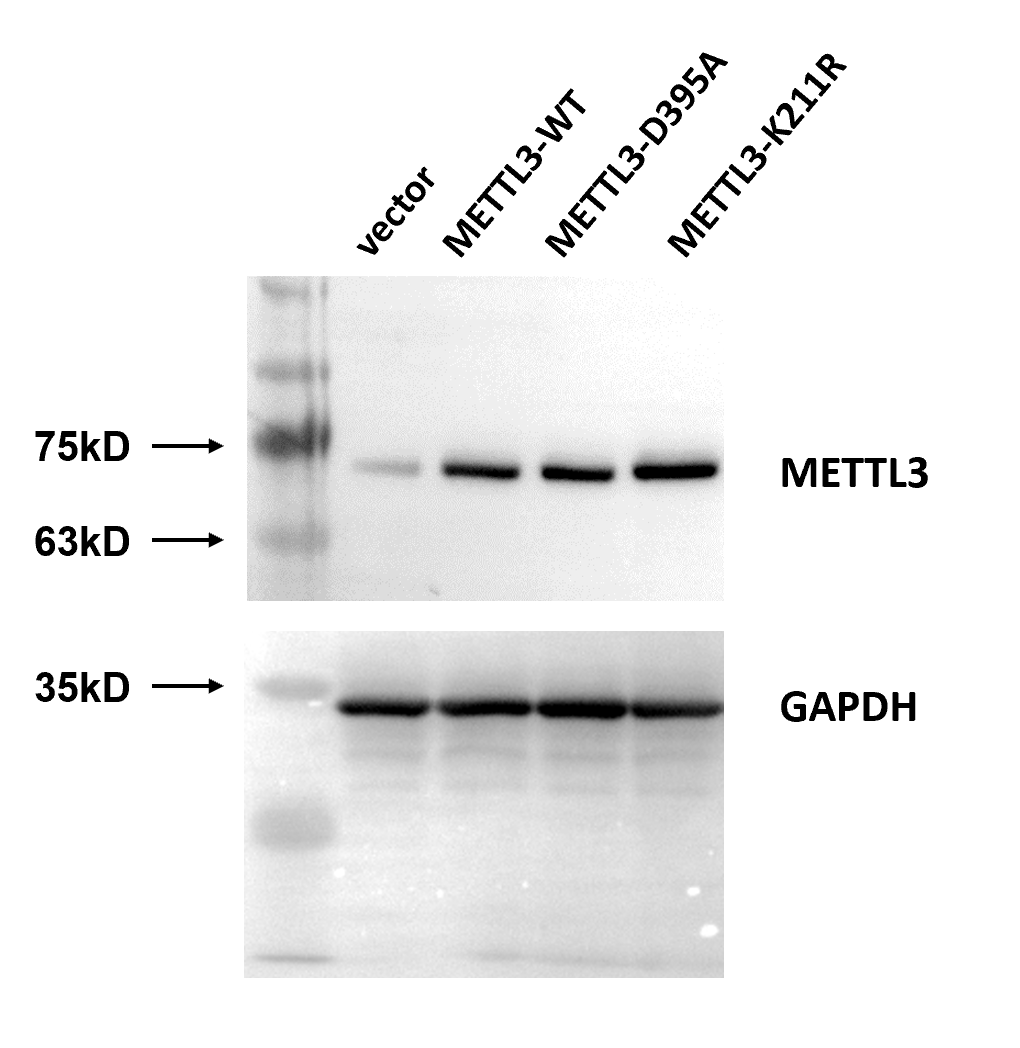


**Figure 3F Protein levels of METTL3 and PLOD2 in Caki-1 cells with overexpression or depletion were determined by western blotting.**


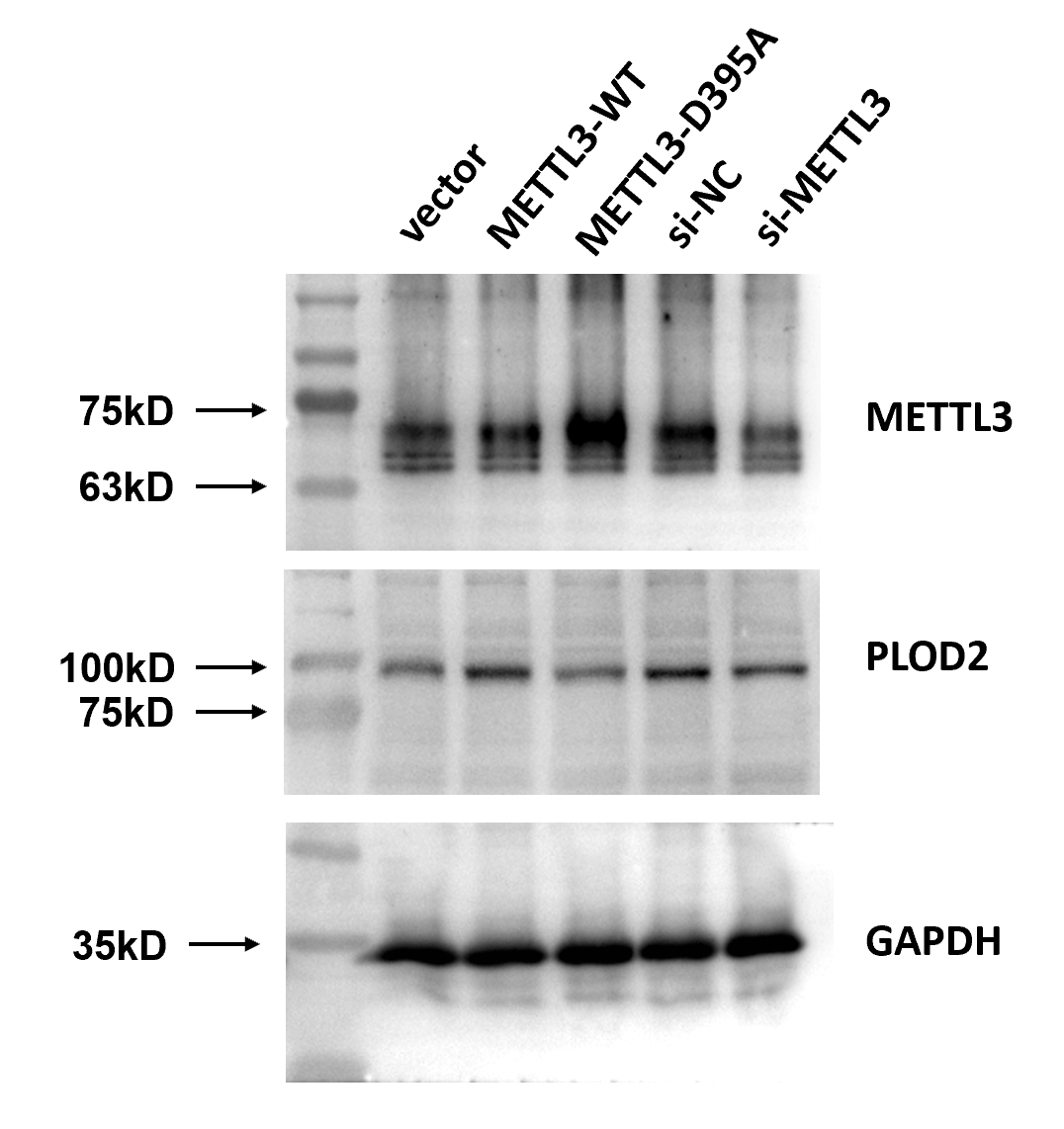


**Figure 4B Expression levels of HIF-1α, HIF-2α, METTL3 and PLOD2 were determined by western blotting after ACHN cells were exposed to normoxia (20% O2) or hypoxia (1% O2).**


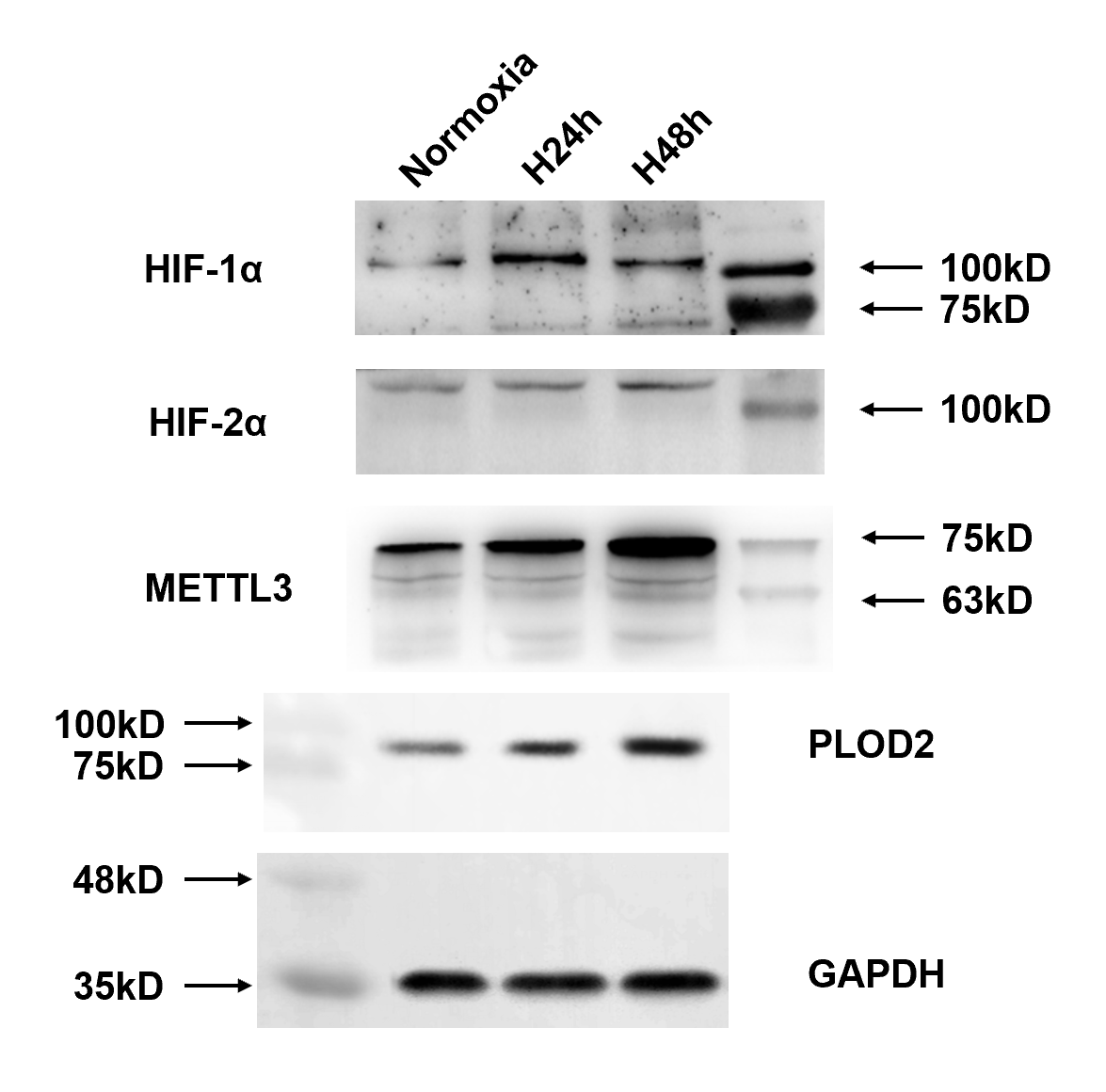


**Figure 4E Examination of the knockdown efficiency when si-HIF-1α, si-HIF-1α, or double knockdown (DKD) were transfected into ACHN cells.**


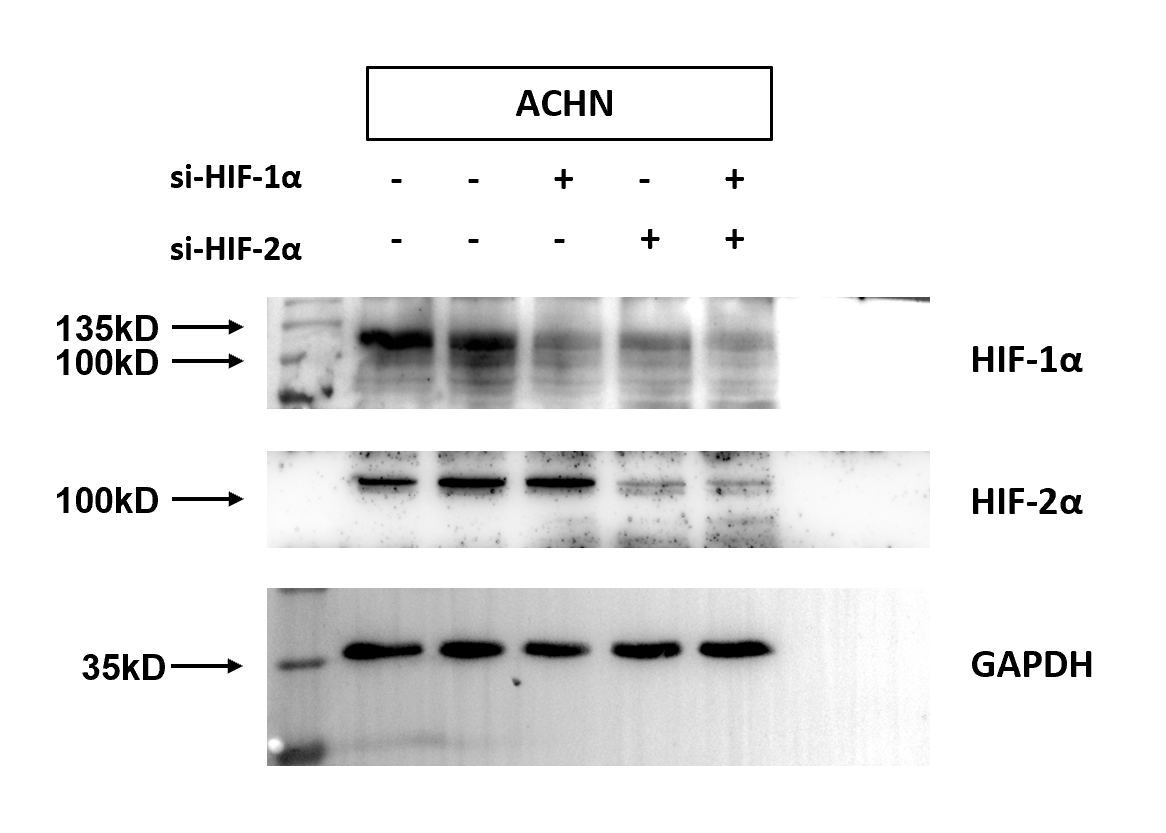


**Figure 4G Silencing of HIFs decreased METTL3 and PLOD2 expression at protein level in RCC cells under prolonged hypoxia.**


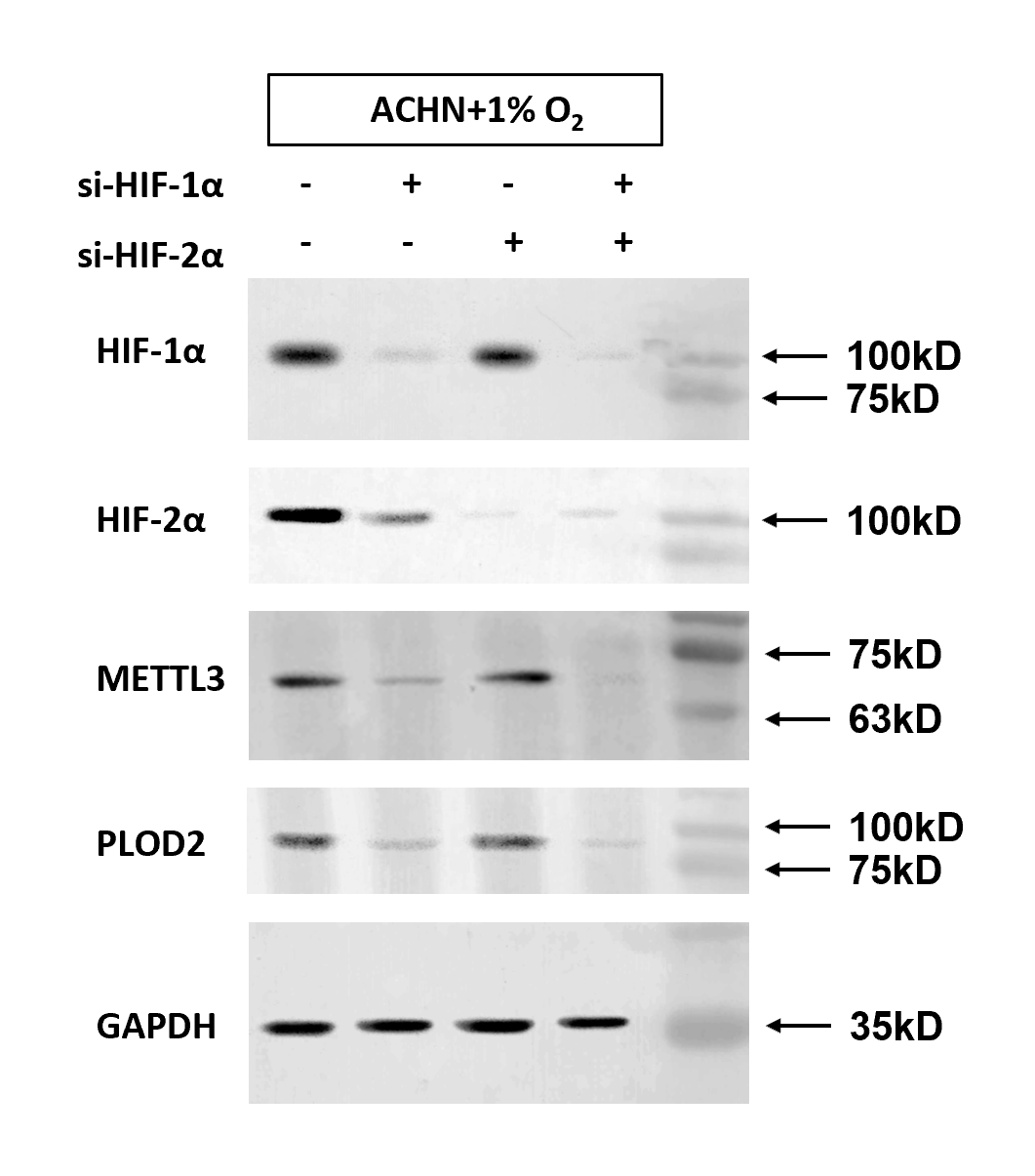


**Figure 4K Expression levels of METTL3 and PLOD2 were examined in ACHN cells by western blotting when prolonged hypoxia was combined with METTL3 silencing.**


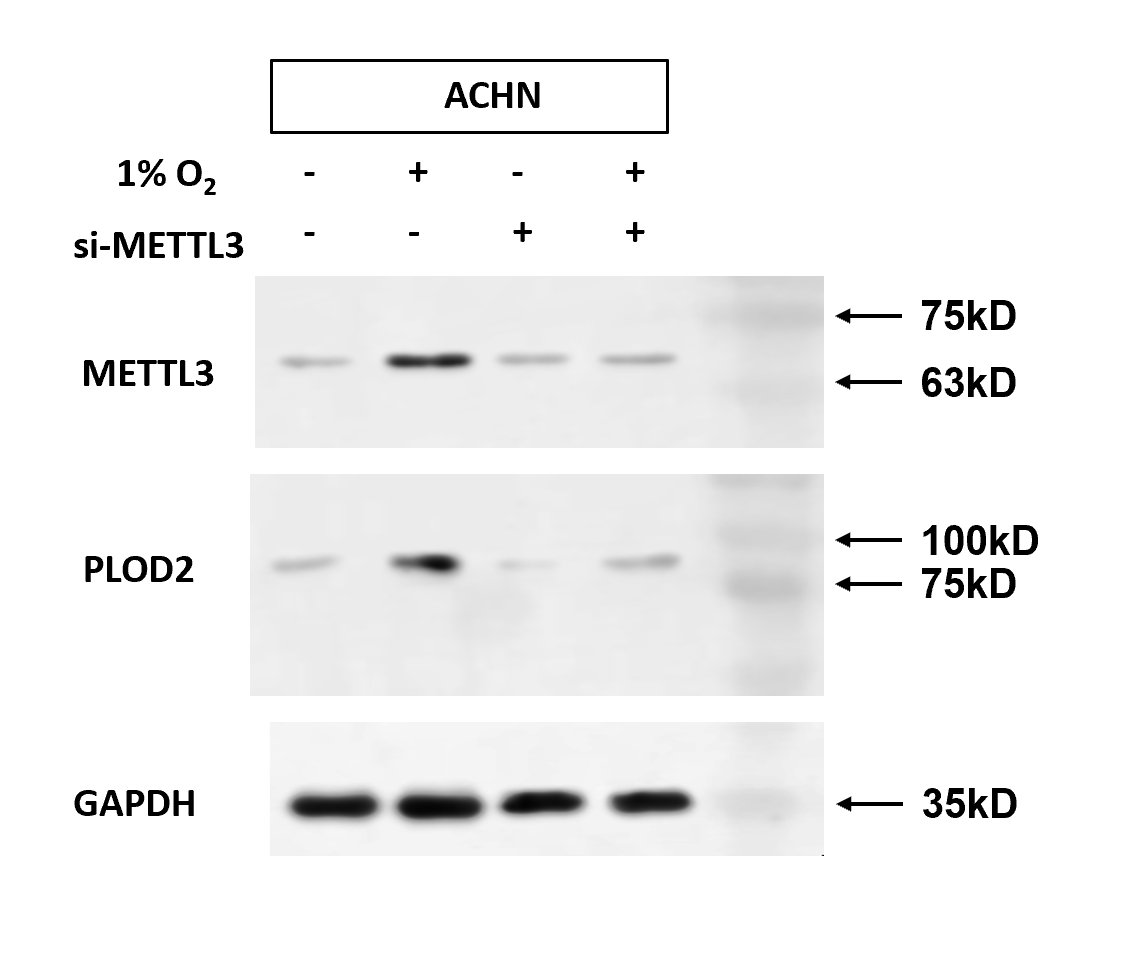


**Supplementary Figure 1A Protein levels of METTL3 and PLOD2 in ACHN cells with METTL3 overexpression or depletion were determined by western blotting.**


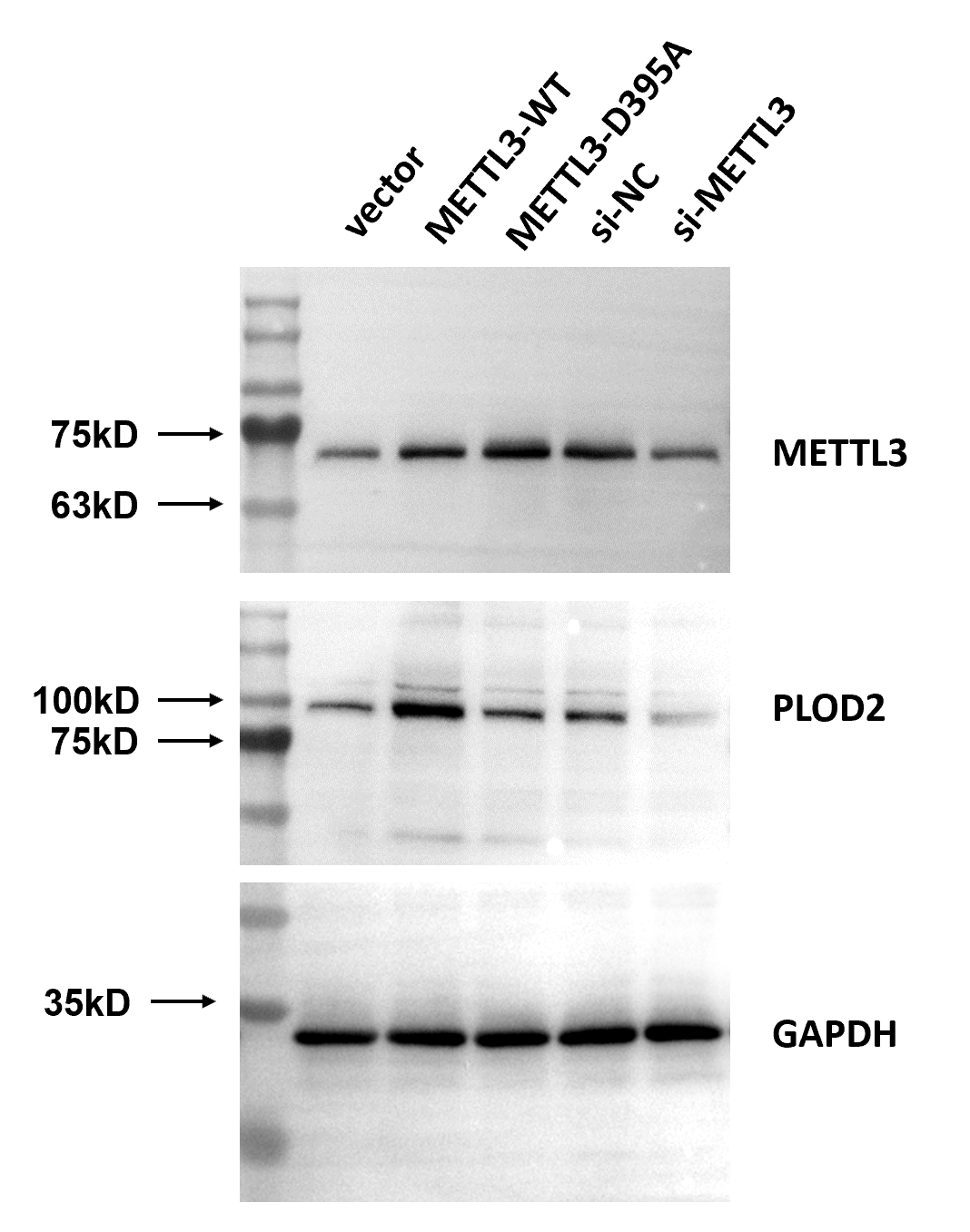


**Supplementary Figure 1D Protein levels of METTL3 and PLOD2 in Caki-1 cells in response to different dose of METTL3-D395A overexpression.**


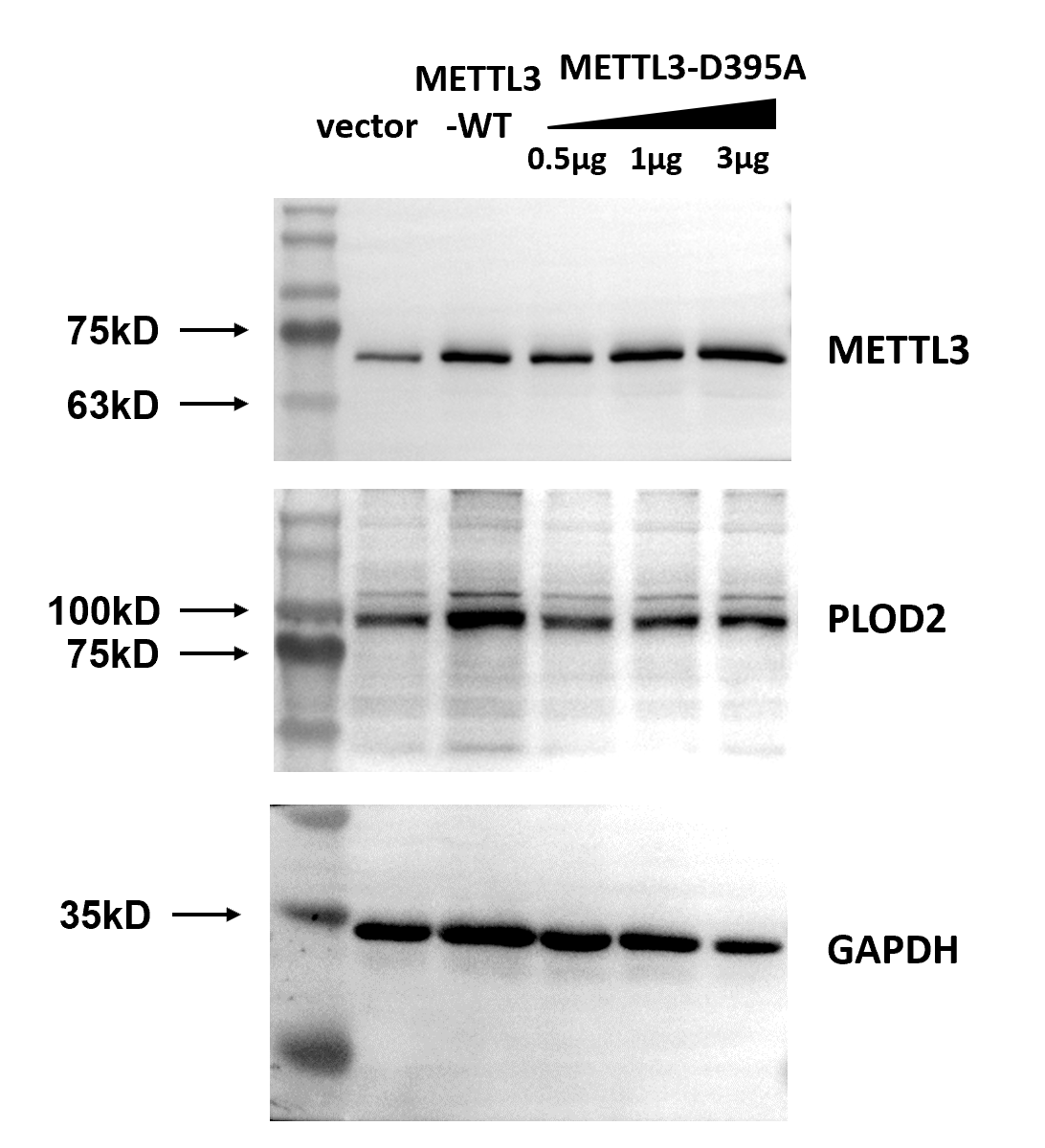


**Supplementary Figure 1G Protein levels of METTL3 and PLOD2 were assessed in Caki-1 cells at different time points following METTL3-D395A overexpression.**


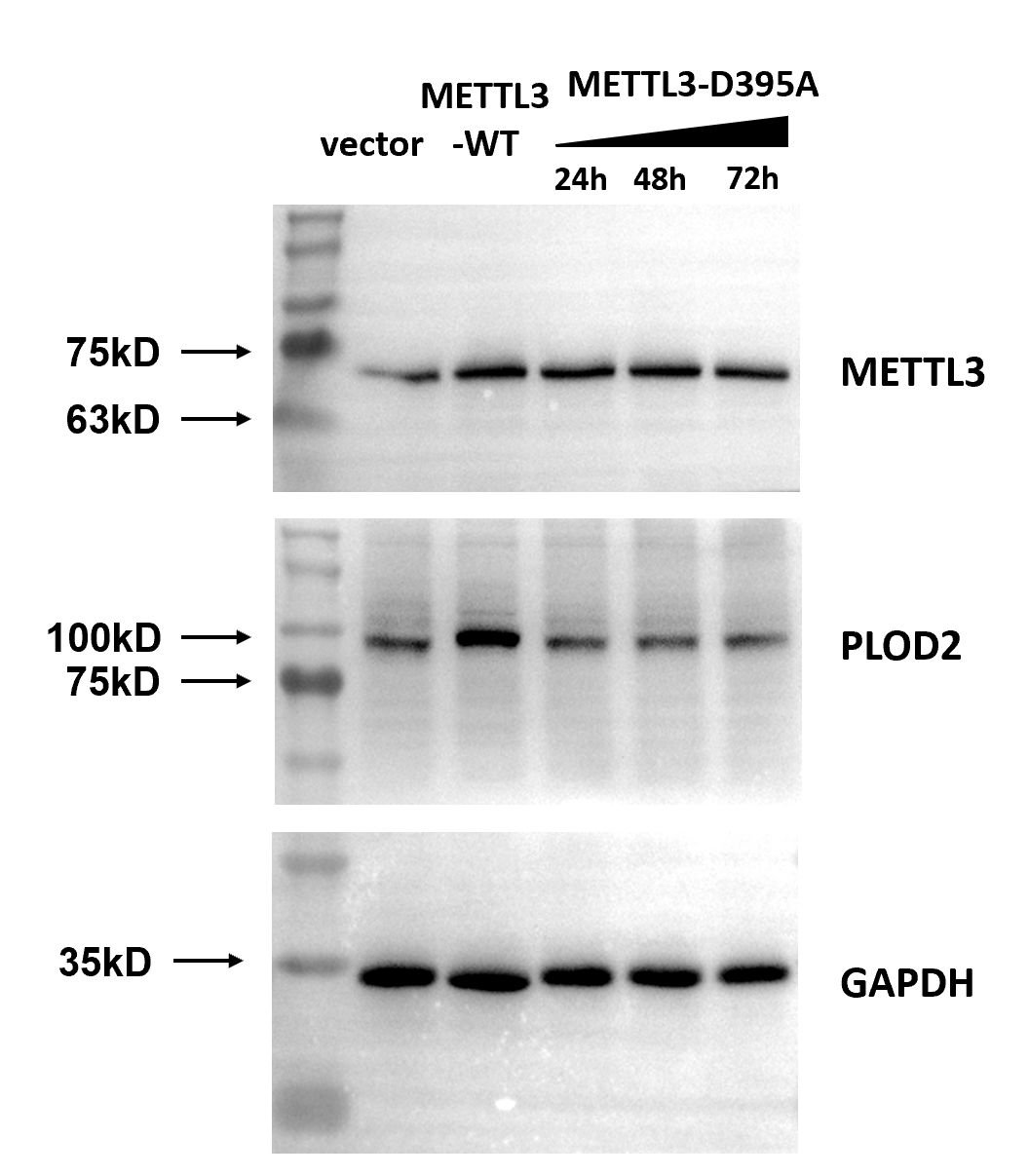

Supplement: Supplementary file 2 — Supplementary material-original western blots [file 41419_2023_6411_MOESM2_ESM.docx]
